# HPV16 and 18 genome amplification show different E4-dependence, with 16E4 enhancing E1 nuclear accumulation and replicative efficiency via its cell cycle arrest and kinase activation functions

**DOI:** 10.1371/journal.ppat.1006282

**Published:** 2017-03-17

**Authors:** Nagayasu Egawa, Qian Wang, Heather M. Griffin, Isao Murakami, Deborah Jackson, Radma Mahmood, John Doorbar

**Affiliations:** 1 Department of Pathology, University of Cambridge, Cambridge, United Kingdom; 2 Francis Crick Institute, Mill Hill Laboratory, The Ridgeway, Mill Hill, London, United Kingdom; University of Wisconsin Madison School of Medicine and Public Health, UNITED STATES

## Abstract

To clarify E1^E4’s role during high-risk HPV infection, the E4 proteins of HPV16 and 18 were compared side by side using an isogenic keratinocyte differentiation model. While no effect on cell proliferation or viral genome copy number was observed during the early phase of either virus life cycle, time-course experiments showed that viral genome amplification and L1 expression were differently affected upon differentiation, with HPV16 showing a much clearer E4 dependency. Although E4 loss never completely abolished genome amplification, its more obvious contribution in HPV16 focused our efforts on 16E4. As previously suggested, in the context of the virus life cycle, 16E4s G2-arrest capability was found to contribute to both genome amplification success and L1 accumulation. Loss of 16E4 also lead to a reduced maintenance of ERK, JNK and p38MAPK activity throughout the genome amplifying cell layers, with 16E4 (but not 18E4) co-localizing precisely with activated cytoplasmic JNK in both wild type raft tissue, and HPV16-induced patient biopsy tissue. When 16E1 was co-expressed with E4, as occurs during genome amplification *in vivo*, the E1 replication helicase accumulated preferentially in the nucleus, and in transient replication assays, E4 stimulated viral genome amplification. Interestingly, a 16E1 mutant deficient in its regulatory phosphorylation sites no longer accumulated in the nucleus following E4 co-expression. E4-mediated stabilisation of 16E2 was also apparent, with E2 levels declining in organotypic raft culture when 16E4 was absent. These results suggest that 16E4-mediated enhancement of genome amplification involves its cell cycle inhibition and cellular kinase activation functions, with E4 modifying the activity and function of viral replication proteins including E1. These activities of 16E4, and the different kinase patterns seen here with HPV18, 31 and 45, may reflect natural differences in the biology and tropisms of these viruses, as well as differences in E4 function.

## Author summary

In HPV induced lesions, the most abundant protein expressed in the productive stage of viral life cycle is E1^E4 (E4), with its expression being coincident with viral genome amplification. To clarify the role of E4 in the high-risk HPV life cycle, we carried out a comparative analysis of E4 function in HPV16 and 18 using an isogenic keratinocyte cell-line background. Our results show that E1^E4 contributes to virus genome replication efficiency and life cycle completion rather than being essential. These effects were seen more dramatically with HPV16. The difference between HPV16 and HPV18 in our system suggests important tropism differences between these viruses. HPV16 E4’s contribution to the virus life cycle is mediated by several activities, including its G2 arrest function, as well as its role in activating members of the MAPK pathway, including ERK, p38, and most notably pJNK. These 16 E4 functions facilitated the nuclear localization of the E1 virus helicase and enhanced E1/E2 dependent viral genome amplification as well as stabilising E2. We suspect that the massive accumulation of E4 in the upper epithelial layers may however underlie a more critical role for E4 post-genome amplification.

## Introduction

Human papillomaviruses (HPVs) are small, non-enveloped DNA viruses that infect cutaneous and mucosal stratified epithelium to induce a wide variety of epithelial lesions ranging from benign papillomas to invasive carcinomas [1]. So far, more than one hundred and fifty HPV types have been completely sequenced [2], with anogenital types being divided into two groups according to their contribution to cancer development. The low-risk types include HPV 6 and 11 that are associated primarily with benign genital lesions. The high-risk types, such as HPV16, 18, 31, 33, and 45 are found in around 99.7% of cervical cancers, with HPV types 16 and 18 being responsible for more than 75% of cervical cancers worldwide [3]. Although both HPV16 and 18 are contained within the Alpha Papillomavirus Genus, they are members of different species (Alpha7 and 9), with different biologies and disease-associations. For all papillomaviruses, the virus life cycle is linked to the differentiation of the infected epithelial cell as it migrates from the basal layer to the epithelial surface. Viral genomes are maintained as nuclear episomes that replicate along with cellular DNA at low copy number in the basal layer. Following cell division, HPV genomes partition into the two daughter cells, with one of these entering the suprabasal layers and committing to terminal differentiation. Although this facilitates expression of L1 and L2 and the assembly of infectious virions, completion of the virus life cycle depends on epithelial site of infection, with life-cycle deregulation leading to neoplasia, and in some instances, the development of cancer [1, 4, 5].

During the HPV life cycle, levels of the viral E1^E4 protein rise as genome amplification begins, allowing accumulation of E4 protein in the upper layer of the epithelium in cells supporting virus synthesis. The E1^E4 protein is translated from a spliced mRNA, with the first 5 amino acids being encoded by the E1 open reading frame [6–8]. The E1^E4 protein associates with and reorganizes the cellular cytokeratin network both *in vivo* and *in vitro* via its N-terminal leucine-rich motif (LLXLL), which may eventually contribute to efficient virus release [9–12]. Recent findings suggest that this interaction may also affect viral genome amplification, with the disruption of the HPV16 E1^E4 keratin binding motif leading to a defect in amplification success during the virus life cycle [13]. Interestingly, over-expression of the E4 protein of HPV1, 16 and 18 in monolayer cell culture induces a robust G2/M cell cycle arrest [14–17], which may create an environment that facilitates efficient E6/E7 driven viral genome amplification. In HPV16, an association of E1^E4 with Cdk1/cyclinB complexes via its PTTP motif is responsible for this effect [15]. Our recent work suggests a further complexity of E1^E4 function, with the protein being modified first by MAPK and then by Cdk1/cyclinB [14, 18] as the infected cell moves from S-phase into G2 prior to growth arrest. These changes modify E4 structure and keratin association, and eventually facilitate E1^E4 cleavage by the protease calpain. This final post-translational modification removes key sequences at the proteins N-terminus, allowing it to multimerise and to be deposited as highly abundant amyloid fibrils [19]. This ordered pattern of expression and protein modification suggests a primary role for E4 in the productive stages of the virus life cycle, including genome amplification, virus assembly, virus transmission and release. Recent data from several groups have suggested a role for E1^E4 in genome amplification and life cycle completion. However, there appear to be significant differences between its roles in different HPV types and when function is examined in different cell backgrounds. Whereas in HPV18 and 31, loss of full length E1^E4 protein led to impaired genome amplification when analysed in human primary foreskin keratinocytes [20, 21], in HPV 11, the truncated E1^E4 has been reported not to compromise life cycle completion, which was examined in N-Tert cells, a human foreskin keratinocyte line immortalized by hTERT (the catalytic subunit of human telomerase) [22]. In HPV16, our previous work showed different effects depending on the length of the E4 protein following expression in NIKS, a spontaneously immortalised human keratinocyte cell line [13]. Taken together, the precise contribution of E1^E4, and the mechanism by which it acts to optimize genome amplification have yet to be firmly established. These varying results do however suggest that E4 may not have precisely the same function in all papillomaviruses, and that this may differ even between members of the high-risk HPV group, mirroring the differences in the functions of other high-risk HPV gene products such as E6 and E7.

To define E4’s role in the virus life cycle more methodically, we have examined its function in the context of the two most important HPV types (HPV16 and 18) during their full productive life cycle. We have taken care to overcome issues relating to keratinocyte batch-variation and/or protocols by using a common cell background (i.e. the isogenic NIKS keratinocyte cell-line), and an optimized organotypic raft culture approach that robustly support the life cycle of a diverse range of HPV types including HPV16 [23, 24]. NIKS (Near-diploid Immortalised KeratinicyteS) is a pathogen-free, immortal human keratinocyte progenitor, that provides a reliable and reproducible model system for the study of epithelial keratinocyte growth and differentiation [25], and the papillomavirus life cycle [26]. Indeed, NIKS epithelium ‘STRATAGRAFT’, is currently being developed by STRATATECH, and is being tested in Phase 3 trials as a temporary skin replacement for burns victims (http://www.stratatechcorp.com/). Based on these systems, we have carried out a comparative functional analysis using genomic mutants, and have in addition made extensive use of time-course experiments to discriminate between loss of function and functional delay in the organotypic raft system. It appears from this that E1^E4 contributes to virus replication efficiency and life cycle completion, rather than being essential for these events, with HPV16 E1^E4 having a much more dramatic contribution when direct comparisons are made in common genetic background. This may be linked to the greater propensity of HPV16 to drive neoplasia at stratified epithelial sites, and thus the greater role for E1^E4 in arresting cell cycle progression in a G2-like state during the late stages of infection. The additional novel observation, that 16E1^E4 prominently sequesters active JNK kinase, and other members of the MAPK family in the cytoplasm during the productive phases of the virus life cycle, provides an additional role of E4 in enhancing the accumulation of the viral E1 helicase in the nucleus. It is clear from this, that 16 E4 contributes to genome amplification success through several molecular mechanisms, and that the extent of E4’s contribution differs even amongst the high-risk HPV group.

## Materials and methods

### Ethics statement

The collection of clinical material used in this study complied with the Helsinki Declaration of 1975, as revised in 1983 as described previously [29]. All tissue sections were obtained as part of a study with Jagiellonian University, Krakow Poland [29], with sample collection being approved by the relevant Ethical Review Board (IRB nr KBET/2/B/2010 dated 07.01.2010). All patients participating in that study were adults and gave written informed consent prior to surgery. All experiments using human tissue are licenced at University of Cambridge by Human Tissue Authority (Licensing number 12196).

### Cell culture and collection of clinical samples

C33a (ATCC), SiHa (ATCC, a cervical carcinoma cell line that constitutively expresses HPV16 E6 and E7), 293T (ATCC) were maintained in Dulbecco’s Modified Eagle’s Medium (DMEM, SIGMA) supplemented with 10% fetal calf serum (FCS, HyClone) and 1% penicillin and streptomycin. NIKS (a gift from Paul Lambert, McArdle Laboratory for Cancer Research, University of Wisconsin), a HPV-negative spontaneously immortalised human keratinocyte cell line, was maintained at sub-confluence on γ-Irradiated J2 3T3 feeder cells (a gift from Paul Lambert) in F medium with all supplements as previously described [27, 28]. All cells were incubated at 37°C in a 5% CO_2_ environment.

### Plasmid construction and site-directed mutagenesis

To construct the HPV18 E4 genomic mutant, a subgenomic HPV18 fragment was excised from plasmid pBSSK-HPV18 (pBSSK-HPV18 was a gift from Craig Meyers, the Pennsylvania State University College of Medicine [30]) by digestion with ApaI, which cleaves at position 663 on pBSSK, and at nucleotide 4535 in the HPV18 genome. This fragment, which contains the E4 ORF, was subcloned into pBC (Stratagene, The Netherlands) and used as a template for PCR-mediated, site-directed mutagenesis (QuickChange XL Site-Direct Mutagenesis system, Stratagene, The Netherlands). Two nucleotide substitutions (T^3467^ to A and T^3470^ to A) were inserted into the E4 ORF using the forward primer 5’-TAT CCG CTA CTC AGC TAG **T**A**A** AAC AGC TAC AGC ACA CCC-3’ and reverse primer 5’GGG TGT GCT GTA GCT GTT **T**T**A** CTA GCT GAG TAG CGG ATA-3’. These changes introduced a translational stop codon into E4, with only a silent change in the overlapping E2 coding sequence. After mutagenesis, the subgenomic fragment was cloned back into pBS-HPV18, and the entire HPV18 genome was sequenced to confirm that the genome contained only the desired mutations. The construction of E4 mutant HPV16 genome (16st15) was described previously [13]. To generate the G2 arrest mutant of HPV16E1^E4, the same W12 strain of the HPV genome (originally cloned into the pSp64 vector, a kind gift from Prof. Margaret Stanley, Department of Virology, University of Cambridge) was cloned into the BamHI site of the modified plasmid pTZh19U (pTZhW12 HPV16 [31].Mutagenesis was performed by PCR-based site-directed mutagenesis as described above (QuickChange XL Site-Directed Mutagenesis system, Stratagene, The Netherlands) using primers AGGCAGCACTTGGCCAATCATTCCGCCGCG and CGCGGCGGAATGATTGGCCAAGTGCTGCCT in order to generate a mutant genome with changes in the 16E1^E4 region necessary for G2-arrest motif. Both wild type (WT) and mutant (T22I, T23I) genomes were sequenced to ensure that no additional base changes were present. The pDrive-GAPDH plasmid used for copy-number evaluation was prepared by cloning a 361 bp fragment of human GAPDH (Genbank accession NM-002046) into pDrive (Qiagen Ltd, UK) after amplification by RT-PCR from total RNA extracted from NIKS (forward primer GCCTCCCGCTTCGCTCTC and reverse primer GCCAGCATCGCCCCACTTG). RNA isolation and RT-PCR were described previously [18]. To construct the E1/E2/E4 expression vector (pIRES-E1E2E4), the region of HPV16 containing the E1, E2 and E4 open-reading frames (ORFs) was amplified from the HPV16 WT and E4KO genome by PCR amplification using primers ACG TGA ATT CTA ATC TAC CAT GGC TGA TCC TGC AGG TAC CAA T and ACG TTC TAG ACG CGG ATC CTC ATA TAG ACA TAA ATC CAG TAG A. The amplified DNA fragments were cloned into the pIRESeGFP (pIRES) vector (Clontech) utilising the EcoRI and BamHI restriction enzyme sites to create plasmids pIRES-E1E2E4 and pIRES-E1E2. The HPV16 origin-containing reporter plasmid (p16Ori-CMV-Gluc) was prepared from pCMV-Gluc2 (New England Biolabs, NEB) by cloning a PCR-amplified HPV16 LCR fragment (nt positions 7267–103) in reverse orientation as shown diagrammatically in S6 Fig (primer sequences available upon request). Ligation was carried out using an In-Fusion Cloning Kit (Clontech) according to the manufacturer’s instructions.

### Generation of NIKS cell lines containing HPV genomes and propagation in organotypic raft culture

Plasmids containing HPV16 and HPV18 genomes (wild type or mutant) were digested with BamHI (HPV16) or EcoRI (HPV18) to release the whole viral genome. The linearized HPV16 or HPV18 genomes were then re-circularized and purified as described previously, before being co-transfected with a plasmid encoding blastocidin into NIKS cells [32]. Twenty-four hours later, NIKS cells were selected with 6 μg/ml blasticidin for 5–7 days, and after screening by PCR and Southern blotting, isogenic WT and E4KO pairs of HPV16 or HPV18 populations with similar viral genomic copy number/cell were used for comparative analysis in organotypic raft cultures as described by Isaacson *et al* [23, 24, 32].

### Differentiation of keratinocytes in semisolid medium

Prior to differentiation, NIKS cells were prepared from monolayer cultures following trypsinization, and suspended in 1.5% methylcellulose as previously described [33]. Approximately 4 x 10^6^ keratinocytes were plated into a total of 6 ml methylcellulose medium in 6 wells of a 24-well plate and incubated at 37°C in a CO_2_-containing humidified incubator for 24–72 h. Cells were washed 5 times by centrifugation in ice-cold PBS and re-suspended in 100 μl Qiagen ATL buffer before DNA preparation using the QIAamp DNA Micro Kit (Qiagen, UK).

### Detection and quantification of DNA

Real-time PCR was used for the detection and quantification of HPV DNA and human GAPDH using the primers and TaqMan probes shown in Table 1. Reactions were prepared in a volume of 25μl containing either 1×TaqMan Universal PCR Mastermix (Applied Biosystems Ltd, UK), 2.5mM of each primer and 6.25mM TaqMan probe, or containing 1× Absolute SYBR Green QPCR Rox Mix (Applied Biosystems Ltd, UK) with 70nM of each primer. PCR was performed using an ABIprism 7500 system with 15 min denaturation at 95°C followed by 40 cycles of 95°C for 15s and 60°C for 60s. For each real-time PCR assay, a standard curve experiment was performed to allow absolute quantification of DNA. For HPV genomic DNA, pTZhW12HPV16 or pBSHPV18 were used as templates, and for human GAPDH, pDriveGAPDH was used as template. Serial dilutions of plasmid DNA were prepared to allow DNA copy number to be plotted against cycle threshold value. All real-time PCR reactions were run in triplicate alongside no-template controls. To quantitate HPV DNA levels in HPV genome-containing cell populations and cell lines, HPV copy number per cell was expressed relative to GAPDH copy number. The human genome blast using designed primers and probe for human GAPDH shown in Table 1 indicated that there are 4 copies, comprising 2 copies of GAPDH and 2 pseudogene copies of GAPDH per NIKS cell. This was confirmed by determination of GAPDH copy number against known NIKS cell number.

**Table 1:** Primer and Probe.

Location	Primer/Probe	Sequence
HPV16 E4	Forward Primer	GACTATCCAGCGACCAAGATCAG
	Reverse Primer	CTGAGTCTCTGTGCAACAACTTAGTG
	TaqMan Probe	CAGACACCGGAAACCCCTGCCAC
HPV18 E4	Forward Primer	CCAGACGTCGGCTGCTACA
	Reverse Primer	GACAGGTCCACAATGCTGCTT
	TaqMan Probe	CCTGGACACTGTGGACTCGCGGA
GAPDH	Forward Primer	CCTCCCGCTTCGCTCTCT
	Reverse Primer	CTGGCGACGCAAAAGAAGA
	TaqMan Probe	TCCTCCTGTTCGACAGTCAGCCGC

### Fluorescence in situ hybridization (FISH)

Slides of formalin fixed, paraffin embedded raft tissue were baked and de-paraffinised by washing three times in xylene followed by two washes in 100% ethanol. The slides were then rehydrated in 80%, 50% and 30% ethanol for two minutes each, and then finally in PBS for 5 minutes. Sections were then heated in 0.01M citric acid (pH 6.0) in microwave for 2 minutes, prior to protein digestion with Digest All 3 pepsin (Zymed) at 37°C for 10 seconds. HPV16 or 18 DNA was labelled with digoxigenin by use of the DIG DNA labeling Kit (Roche Applied Science Gmbh, Germany) according to the manufacturer’s instructions. DIG-Labeled HPV DNA was added to slides, denatured for 5 min at 76°C, and incubated overnight at 42°C. After washing, the DIG-positive HPV probe was detected with an anti-digoxigenin-POD antibody at 1:400 (Roche Applied Science Gmbh, Germany) and the signal amplified using a Tyramide Signal Amplification Kit (Perkin-Elmer Ltd, UK) according to the manufacturer's instructions. E1^E4 protein and nuclei were identified with anti-16E1^E4 antibody TVG 405 conjugated to Alexa 488 and DAPI (4', 6-diamidino-2-phenylindole).

### Immunofluorescence and immunohistochemistry

**Fig 1:**
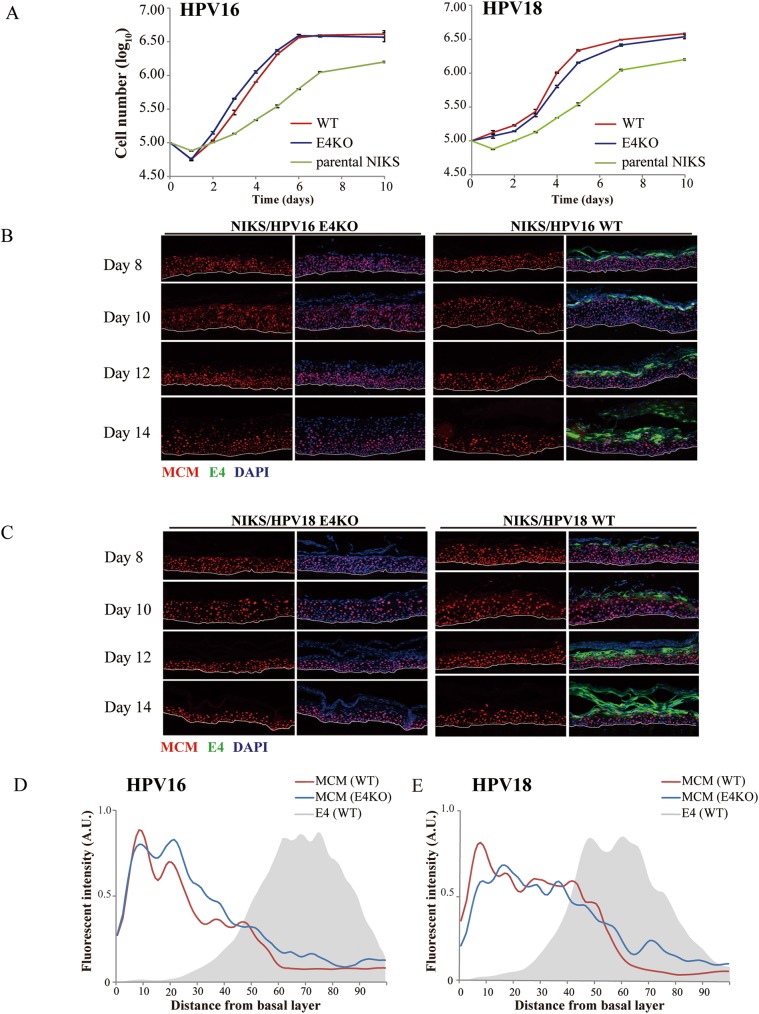
Loss of full length E1^E4 does not affect NIKS cell growth or the early stages of either the HPV16 or HPV18 life cycle. **(A)** Cell growth in monolayer culture was monitored over a 10 day period after plating NIKS cells harbouring the WT or E4KO genomes at the same cell density. Although both HPV16 and 18 increase the growth rate of NIKS, no difference was apparent when WT and KO genomes were compared. The data are plotted as log_10_ cell number per well (averaged from triplicate experiments) against time (days). **(B)** Raft tissue derived from NIKS cells harboring HPV16 WT and E4KO genomes was harvested at 8, 10, 12 and 14 day time-points post-differentiation prior to staining for E1^E4 (green), MCM (red) and DNA (blue; DAPI). MCM was used as a surrogate maker for the expression of viral E7 protein. The dotted lines indicate the position of the basal layer. Images were captured using a 10x objective. **(C)** Raft tissue derived from NIKS cells harboring HPV18 WT and E4KO genomes stained as in (A). **(D & E)** The extent to which cells were driven into cycle in NIKS cells harbouring WT or E4KO was determined by measuring the extent and intensity of MCM staining from the basal layer to rafts surface (see materials and methods) for the HPV16 (C) and 18 (D) WT or E4KO raft tissues at day 14 post-differentiation. The distribution and intensity of E1^E4 in the WT rafts is shown as the grey shadow. No consistent differences were apparent during the early stages of the virus life cycle.

Immunofluorescence and immunohistochemistry were performed as described previously [10]. For activated MAPK detection, the formalin fixed, paraffin embedded tissue sections were incubated in solution D pH 9.0 (Dako, Glostrup, Denmark) for 10 min at room temperature prior to autoclaving for 2 min at 121°C. The antibodies used were anti-phospho-ERK1/2 (Thr202/Tyr204) rabbit mAb (Cell Signaling Technology), anti-phospho-p38MAPK (Thr180/Tyr182) rabbit mAb (Cell Signaling Technology), anti-phospho JNK rabbit polyclonal antibody (abcam, UK), anti-MCM2 rabbit polyclonal antibody (ab31159, abcam, UK), anti-HA (16B12; Covance, Princeton, NJ) and anti-16E1^E4 antibody TVG 405 [34] conjugated to Alexa 488 and an anti-E2 rabbit polyclonal antisera ([35]; a gift from Dr Yuezhen Xue, Institute of Medical Biology, Singapore). To measure the intensity and distribution of the stained protein by ‘cross-sectional imaging’, digital images of immuno-fluorescently stained raft tissues were captured with a DeltaVision microscope and imaging system (GE Healthcare Life Sciences) fitted with a 10x objective or using a Panoramic Slide Scanner, 3D Histotech, UK. The captured images were then analysed using ImageJ in order to establish the intensity of staining through the epithelial layers, from the basal layer to the cornified layer. For each immunofluorescent stain, these ‘top to bottom’ scans were collected across 10 areas of equal thickness that were derived from at least 3 different raft sections. Each of the graphs shown (e.g. in Fig 1D and 1E) represents an average of at least 100 individual cross-sectional profiles therefore, and is in general more representative of the raft phenotype than the individual IF image. Where the tissue architecture of the raft was disrupted during the tissue sectioning (e.g. image shown in Fig 1C (NIKS/HPV18 WT day 14), gaps in the continuity of the scan were compensated for during the analysis. In most cases, such disruption was minimised by positioning blocks in the microtome so that the sectioning blade traversed the block from the epithelial surface towards the epithelial basal layer. Because E4 can disrupt cell-cell contacts, such disruption to raft structure was more obvious with the WT rafts and also at late time points.

### Western blotting analysis

Proteins were extracted from tissue sections as previously described [36] and quantified using the BCA protein assay kit (Pierce, UK), before being separated on 4–12% gradient polyacrylamide-SDS-Tris-Tricine denaturing gel (Invitrogen, UK) and transferred onto PVDF membranes (Bio-Rad). After transfer, membranes were blocked for 1 hour at room temperature in 1% milk in PBS-T (PBS, 0.1% tween20). Blots were then incubated overnight at 4°C with the appropriate primary antibody diluted in 1% milk PBS-T. Primary antibodies used were anti-E2 rabbit polyclonal antisera [35], anti-tubulin clone B512 (Sigma, UK), anti-HPV16L1 (CAMVIR-1, Santa Cruz, USA), anti-GFP clone B-2 (Santa, Cruz), anti-16E1^E4 antibody TVG 405 or anti-GAPDH clone 374 (Chemicon, UK), followed by the appropriate HRP-conjugated secondary antibody (GE Healthcare, UK), and detection using ECL, or ECL plus kits (GE Healthcare, UK) or by the appropriate IRDye 800CW fluorescent secondary antibody (Licor, UK) followed by detection using an Odissey imaging system (Licor, UK).

### Vector construction and retroviral infection

The production and infection of recombinant lentiviruses and retroviruses were accomplished as previously described [37]. Lentivirus or retrovirus vectors CSII-TRE-Tight-HA16E1, in which the N-terminal hemagglutinin (HA)-tagged codon-optimized HPV16 E1 was inserted under a tetracycline-responsive promoter, and pQCXIZeo-tetON ADV was described previously (the virus vectors were a gift from Tohru Kiyono, National Cancer Center Research Institute, Tokyo, Japan [38, 39]). To introduce mutations into the HPV16E1 MAPK phosphorylation sites, PCR-based mutagenesis was used with the KOD-plus mutagenesis kit (Toyobo, Japan), with all mutants being completely sequenced to ensure that no additional changes had been introduced. To generate SiHa cells expressing HA-HPV16E1 in a doxycycline-inducible manner, SiHa cells were seeded 1 day before and inoculated with pQCXIZeo-tetON ADV at MOI of 3 followed by zeocin selection (2 μg/ml). The generated SiHa-tetON cells were then infected with CSII-TRE-Tight-HA16E1 (SiHa-tetON/CSII-TRE-tight-HA16E1). The expression of E1 was induced in the presence of 1 μg/ml of Doxycycline.

### Recombinant adenovirus (rAd) infection and plasmid transfection

Recombinant adenoviruses (rAd) expressing 16E1^E4 (rAd16E1^E4) or β-galactosidase (rAdβ-Gal) have been described previously [15, 40]. For infection experiments, 1x10^6^ SiHa cells were seeded into 90mm tissue culture dishes, or 1x10^4^ SiHa-tetON/CSII-TRE-tight-HA16E1 cells were seeded into Falcon 4 well culture slides (BD Biosciences, USA). 24h after seeding, either rAd16E1^E4 or rAdβ-Gal was added to the media at a multiplicity of infection (MOI) of 100. Cells were subsequently harvested at 24 or 48 h post-infection unless otherwise stated. Transfection was performed using the Effectene Transfection Kit (Qiagen, UK) according to the manufacturer’s protocol. The HPV16 E5-expressing SiHa cell line was established as described previously [18].

### Transient replication assays

Cells were seeded at a density of 4x10^5^ cell per 60mm dish, and were transfected with pIRES-E1E2 or pIRES-E1E2E4, and an HPV16 origin-containing plasmid (p16Ori), a kind gift from Peter Howley (University of Harvard, USA) [41], using the Effectene transfection reagent (Qiagen) according to the manufacturer’s instructions. The cells were harvested as for Western blotting (above), and low molecular weight DNA was extracted as previously described [42] and purified using Wizard spin columns (Promega). To distinguish replicated DNA from input DNA, the extracted plasmid samples were compared before and after DpnI digestion by Southern blotting as described by Del Vecchio et al [41]. To linearize p16Ori, the sample DNA was digested with XmnI. The digested samples were separated by 1% agarose gel electrophoresis and then analysed by Southern blotting essentially as described previously [41]. To measure the relative replication activity, the amount of amplified DNA was normalized to the signal of the input p16Ori DNA for each experiment. Hybridisation signals were quantitated using a Phosphor Imager (Storm) and Image Quant 5.0 software.

For the Luciferase DNA replication assays, NIKS cells were seeded at a density of 2x10^5^ cells per well into 6-well plates without feeder cells, before being transfected using FuGENE HD (Fisher Scientific, UK) with 2ug of pIRES-E1/E2 or pIRES-E1/E2/E4, along with 15ng of the HPV16 origin-containing reporter plasmid (p16Ori-CMV-Gluc) and 15ng of pCMV-Cluc2. 12 hours post-transfection, the media was replaced with F medium with all supplements and feeder cells were added. 48 hours post-transfection, the media was replaced. 24 hours later, culture media was collected and luciferase activity measured using a BioLux Gaussia Luciferase Assay Kit and a BioLux Cypridina Luciferase Assay Kit (NEB, UK). Gaussia Luciferase activity was normalized against Cypridina Luciferase activity.

### Southern blot analysis

Total genomic DNAs were isolated using QIAamp DNA blood mini kit. Total genomic DNAs isolated from each population were digested with either BamHI or HindIII for at least 8h at 37°C and electrophoresed on a 0.8% agarose gel. Total genomic DNA isolated from untransfected NIKS cells was used as a negative control. Following electrophoresis, DNAs were transferred to a nylon membrane (Hybond-N; Amersham, Piscatway, NJ) and hybridized to [α-32P]dCTP-radiolabeled DNA probe for full-length HPV16 prepared with Ready-to-Go DNA labelling system (GE Healthcare Life science). Radioactive bands were detected using a Storm imaging system (Amersham).

### Northern blot analysis and amplification of papillomavirus oncogene transcripts (APOT) assay

Total RNAs from cells (C33a, SiHa) transfected with pIRES-16E1/E2/E4, pIRES-16E1/E2 or pIRES (empty plasmid) were extracted using RNAeasy mini Kit (Qiagen). RNA samples were electrophoresed on a 1.2% formaldehyde agarose gel. Following electrophoresis, RNAs were transferred to a nylon membrane (Hybond-N; Amersham, Piscatway, NJ) and hybridized to [α-32P]dCTP-radiolabeled DNA probe for E1E2E4 DNA fragment of HPV16 prepared with Ready-to-Go DNA labelling system (GE Healthcare Life science). Radioactive bands were detected using a Storm imaging system (Amersham). APOT assay using RNA sample of NIKS/HPV16 was carried out following the protocol described previously [43].

### Detection and quantification of viral gene expression by qPCR

Total RNAs from NIKS containing HPV genome cell were extracted using RNAeasy mini Kit (Qiagen). Total RNA (1 μg) was reverse transcribed with 100 U of SuperScript III Reverse Transcriptase (ThermoFisher scientific) using 100 μM random hexamer primers according to the manufacturer’s instructions. Reactions were prepared in a volume of 20μl containing either 1×T ABsolute qPCR SYBR Green Mixes (ThermoFisher scientific) with 70nM of each primer. Each primer set were designed to detect the viral gene transcripts specifically (the primer sequences are available upon request). PCR was performed using a ViiA 7 system (ThermoFisher scientific) with 15 min denaturation at 95°C followed by 40 cycles of 95°C for 15s, and 60°C for 60s. For each real-time PCR assay, a standard curve experiment was performed to allow absolute quantification of cDNA.

## Results

### Loss of full length E4 does not affect the early stages of either the HPV16 or HPV18 viral life cycle

The role of E1^E4 in the virus life cycle has been examined in different HPV types by different groups using different cell backgrounds and protocols [13, 20–22], prompting us to carry out a comparative analysis in the same cell background. To do this, a ‘generic’ keratinocyte cell line that supports the life cycle of a range of different HR HPV types was used [23, 24], in conjunction with a common protocol for the transfection and maintenance of genome-containing cells. As reported previously, this approach allows the generation of cell lines harboring HPV genomes as viral episomes, with no obvious signs of viral genome integration, either by Southern blotting or APOT analysis (see [23] and S1 Fig). Blotting was carried out after blastocidin selection and the expansion of HPV-containing cell populations, or after the isolation and expansion of HPV-containing cell clones. In all cases, supercoiled, relaxed (open) circular, and linear HPV genomes were detected, with a prominent 8Kb band being apparent after restriction enzyme digestion using a single-cut restriction enzyme. Results from the analysis of three clonal populations (T1, T2 and T3) are shown in S1A Fig as an example. The E6/E7 mRNA expression pattern visualized in these cell lines by APOT assay indicates that these genes are expressed from episomal rather than integrated viral genomes and is shown in S1B Fig, (tracks T1, T2 and T3). The aberrant pattern seen in a cell line harboring integrated HPV genomes is shown in the lane marked ‘IN’, with the range of episomal copy numbers seen in individual clones being shown for HPV16 and 18 in S1C and S1D Fig. Although individual clones, (which represent expansion from single cells in the cell population) contained a range of genomic copy numbers, viral episomes were always apparent. When making comparisons, we were careful to adjust our transfection to achieve the same average copy number in the cell population, or to use copy-number matched clones. The HPV16 E4KO mutant used in this study contained a substitution of T^3384^ to A, in order to introduce a stop codon at amino acid position 15 in the HPV16 E4 protein sequence. In the context of the HPV16 genome, this is the shortest E4 truncation that is silent in the E2 reading frame. Although close to the E1^E4 slice acceptor site at nucleotide 3357, the T^3384^ to A base substitution did not affect the relative abundance of viral transcripts spanning E2 or E1, or the use of the E1^E4 splice site (S2A and S2B Fig). Cell lines harbouring this mutant genome as a viral episome were successfully established, as described previously by Nakahara and colleagues [13]. In HPV18, the E4KO mutant was made by double point mutations of T^3467^ to A and T^3470^ to A, which introduces two stop codons at position 17 and 18 of the E4 coding sequence without changing the coding capacity of the E2 ORF. Comparable with the results from the HPV16 analysis, no changes in patterns of transcription were seen. NIKS populations and clonal cell lines containing the wild type (WT) and E4KO genomes of both HPV types were subsequently prepared. Interestingly, the average genome copy in transfected NIKS population was often higher for HPV18 (approximately 200 copies/cell) than HPV16 (approximately 100 copies/cell), and while individual clones derived from each transfected NIKS population exhibited a range of episomal copy numbers (see S1C and S1D Fig), no significant differences were seen between NIKS cells harbouring WT or E4KO genomes, allowing isogenic WT or E4KO cell line pairs with similar copy numbers to be selected for comparative analysis. To generate cell populations with matched copy number, we found that a lower level of HPV18 genomic DNA was generally required at the transfection stage.

Previous studies have suggested that loss of full length HPV18 E1^E4 can confer a growth advantage on undifferentiated primary human foreskin keratinocytes [21]. To look at this further, HPV16 or 18, copy-number matched WT or E4KO-containing NIKS were plated, and grown in monolayer culture, from day 1 to day 10 (Fig 1A). NIKS were harvested after removal of feeder cells, and counted using a Z1 Coulter Particle Counter (Beckman Coulter, California, United States). Monolayer tissue culture approximates the epithelial basal layer, especially at confluence when cells become contact inhibited but have not begun to differentiate [23]. Such cells lack detectable E4 by immunostaining. As shown in Fig 1, the WT and E4KO genomes of both HPV16 and 18 confer a growth advantage on NIKS keratinocytes when compared to parental cells. However, no significant and/or reproducible difference was apparent between WT and E4KO genomes of either HPV16 or 18. When taken together, these results indicate that the E1^E4 protein does not obviously contribute to early events in the HPV life cycle, such as the initial amplification following infection, the maintenance replication of the viral genome or the regulation of the viral proteins that drive cell proliferation in the basal layer.

We next considered whether this lack of an obvious E4 function in basal cells extended to the basal and parabasal layers when cells were propagated in organotypic raft culture. Raft culture provides a spatial separation of cells at various stages of differentiation, and closely mimics the differentiation and organization of normal epithelial tissues. Rafts were harvested at 8, 10, 12 and 14 days after placing the cell monolayer at the air/liquid interface in order to examine the consequences of E4 loss as the extent of differentiation increases. As expected, E4 was not detectable in the lower epithelial layers by immunofluorescence staining at any of the time points, but was apparent in the upper epithelial layers in the WT rafts (Fig 1B and 1C, right panels), becoming more extensive as the extent of differentiation increased from day 8 to 14. The extent of cell cycle entry in basal and parabasal layers was examined by staining for the cellular MCM protein. MCM can be regarded as a surrogate marker of E7 expression when present above the basal layer in HPV-driven rafts [23], with expression declining as the extent of differentiation increased from day 8 to 14. Because of this, raft tissues of similar thickness, and which exhibited a similar degree of differentiation were chosen for comparison. As shown in Fig 1B and 1C, no obvious differences in MCM staining were apparent between WT and E4KO rafts at any of the time points examined, with broadly similar results for HPV16 and 18. To rule out the possibility that E4 may affect either the density of MCM-positive cells in the lower epithelial layers, or the intensity of staining in each cell, HPV 18 WT and E4KO rafts were digitally scanned from basal layer to the top and analyzed by cross-sectional imaging analysis (Fig 1D and 1E). Again, no obvious difference was apparent in the MCM expression profile in the lower epithelial layers, supporting the conclusion that the early stages of the virus life cycle are not dependent on HPVs ability to express E4. A similar observation was previously reported using an *in vivo* model of E4 function and neoplastic progression in the CRPV/domestic rabbit system [44].

### HPV16 and 18 genome amplification is delayed rather than abolished in the absence of E4 following differentiation

**Fig 2:**
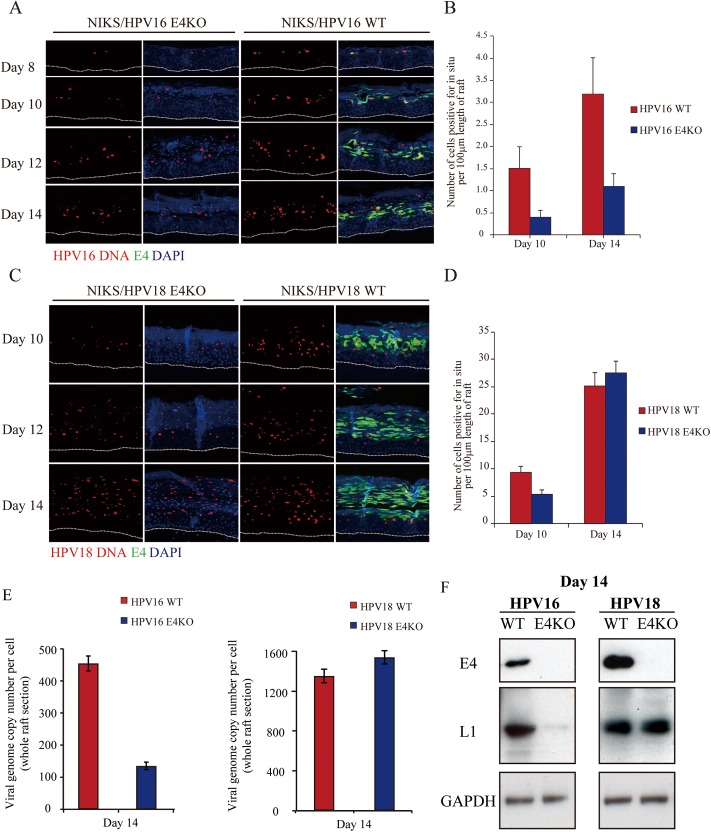
Differentiation dependent HPV genome amplification is delayed rather than abolished in the absence of E1^E4. **(A)** Viral genome amplification after differentiation was detected by *in situ* hybridization (red) in raft tissues generated from NIKS cells harboring HPV16 WT or E4KO genomes. Time post-differentiation is indicated in days on the left. Staining for E1^E4 and DNA (DAPI) are shown as green and blue. The dotted line indicate the position of the basal layer. Images were captured using a 10x objective. **(B)** The average number of cells showing evidence of HPV16 viral genome amplification as revealed by *in situ* hybridization were counted from 10 raft sections of comparable thickness in the NIKS HPV16 rafts. **(C)** Viral genome amplification after differentiation was detected as described in (A) above, although in this case, rafts were derived from NIKS harboring HPV18 WT or KO genomes. **(D)** The average number of cells showing evidence of HPV18 viral genome amplification were counted as outlined in (B) above. **(E)** Total DNA was extracted from microdissected raft epithelium (WT or E4KO) at the 14 day time point, and total HPV16 (left) and HPV18 (right) genomic DNA was quantified by qPCR. **(F)** Total protein was extracted from microdissected raft epithelium (WT or E4KO) at the 14 day time point, and the levels of E4 and L1 protein visualised by western blotting.

During the HPV life cycle, viral genome amplification begins during host epithelial differentiation, and is coincident with E1^E4 expression [6, 31]. Indeed, the role of E1^E4 in viral genome amplification has been examined using a number of HPV genomic mutants in different cell backgrounds using a range of differentiation protocols [13, 20–22]. Despite differences in the extent of productive infection in these systems, an almost complete failure to support genome amplification was reported for HPV18 and 31 [20, 21], with no effect on genome amplification reported for HPV11 [22]. Here we have focused specifically on the two most important high-risk HPV types (HPV16 and 18) using a common isogenic cell background, with differentiation being carried out over an 8–14 day time course. We conclude from this more extensive analysis, that E4 is not in fact essential for viral genome amplification, as *in situ* hybridization signals were apparent in the middle and upper layers of both WT and E4KO mutants in both the HPV16 and 18 rafts (Fig 2A and 2C). While viral genome amplification can thus be triggered without E4 proteins, its detection by *in situ* hybridization was however much reduced in HPV16 E4KO raft tissues at all time-points (Fig 2A), and was much more apparent when the extent of differentiation in the raft was limited (i.e. day 8 and 10). The loss of E4 had no apparent effect on differentiation however, as revealed by hemotoxylin and eosin staining (H&E), and the detection of early and late differentiation markers (keratin 10 and filaggrin) (S3 Fig). In fact the raft time course experiments described here may explain the heterogeneity of results reported previously, with E4 effects becoming less apparent as the extent of epithelial differentiation increases over time. To examine this more precisely, the 10 and 14 day raft time-points were digitally scanned (Panoramic Slide Scanner, 3D Histotech, UK), and the number and intensity of the HPV *in situ*-positive cells averaged across 10 raft sections of comparable thickness. Using this approach, which overcomes some of the limitations of showing just selected images, the extent of genome amplification was found to be around three fold higher in the NIKS/HPV16 rafts when compared to the NIKS/HPV16 E4KO rafts at both the 10 and 14 day time points. Comparable raft cultures prepared with the HPV18 E4KO NIKS showed a much less dramatic reduction (Fig 2C). At day 10 and 12, viral genome amplification was lower with the E4KO mutant than with the WT genome, but by day 14, viral genome amplification was similarly extensive in both the WT and E4KO genomes (Fig 2D). Although such *in situ* methodologies are particularly useful in understanding events in heterogeneous tissues such as rafts (Fig 2B and 2D), we went on to also examined raft sections by qPCR and western blotting following laser capture microscopy (LCM) and/or epithelial microdissection (Fig 2E and 2F). Similar to what was seen in the *in situ* hybridisation studies (Fig 2A–2D), the effect of E4KO on genome amplification (Fig 2E (and L1 accumulation (Fig 2F)) was again seen much more clearly with HPV16 than HPV18. When taken together, these results indicate that for both HPV16 and 18, genome amplification is restricted rather than abolished in the E4KO cells upon differentiation, with apparently similar levels of genome amplification being seen irrespective of E4 presence in the mature HPV18 rafts.

### Capsid protein expression appears linked to genome amplification success, and is not necessarily compromised by E4 loss

**Fig 3:**
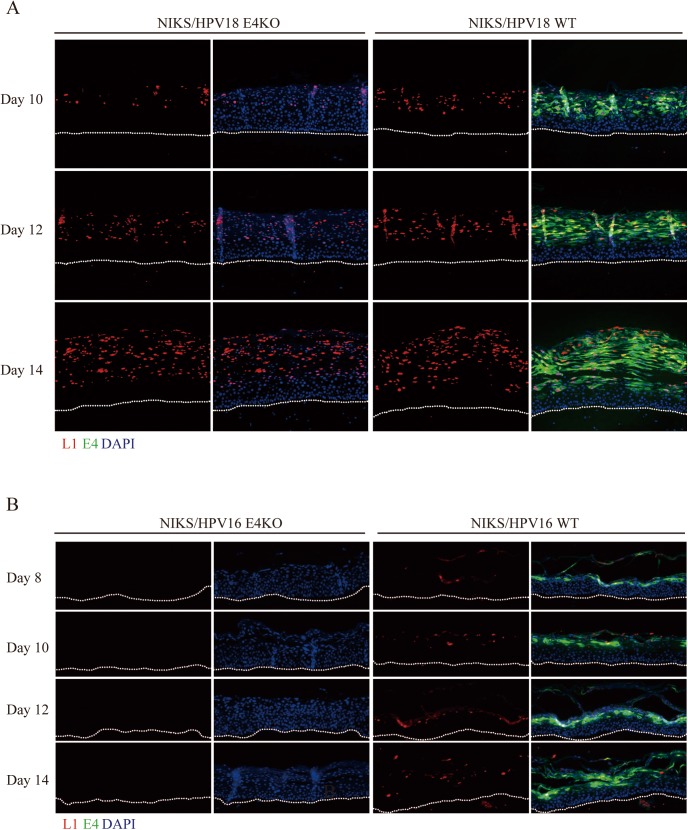
Viral capsid protein expression appears linked to viral genome amplification success in HPV16 and 18 rafts. Raft sections derived from NIKS cells harboring HPV18 **(A)** or HPV16 **(B)** WT or E4KO genomes were analyzed at different time points post-infection, before being stained for E1^E4 (green), L1 (red) and DNA (blue; DAPI). The dotted lines indicate the position of the basal layer. Images were captured using a 10x objective.

The final stages of the HPV productive cycle involve expression of the viral capsid proteins (L1 and L2), to allow genome packaging. To address whether the absence of E1^E4 directly compromises the expression of capsid proteins *per se*, we compared the extent to which L1 accumulation is dependent on E4 in HPV16 and HPV18 rafts during our time course. Indeed, this was initially suggested from the analysis of total protein extracts prepared from SDS-solubilised, microdissected day 14 raft epithelium shown in Fig 2F, where the HPV16 E4KO was found to support L1 accumulation only poorly. When L1 distribution was examined, the extent of L1 expression was found to be lower than WT in the HPV18 E4KO rafts at day 10 and 12, equivalent by day 14, when differentiation was more extensive (Fig 3A). This pattern is very similar to that seen for viral genome amplification, suggesting that genome amplification and capsid synthesis are coordinated events. The similarities seen between the day 14 rafts suggest that 18 E1^E4 does not play an essential role in either the regulation of genome amplification or the expression of virion structural proteins (Fig 2C, Fig 3A). By contrast, no detectable L1 was found in raft tissues containing HPV16 E4KO mutants by immunofluorescence (Fig 3B), only barely detectable levels of L1 transcripts and L1 protein detectable by Western blotting (Fig 2G). During the course of our experiments, three different HPV16 E4KO populations were examined, as well as one clonal cell line harboring the HPV16 E4KO genome, but on no occasion did we detect any expression of 16L1 by immunofluorescence staining. In our experience, HPV16 NIKS are always less proficient in supporting the full productive life cycle than HPV18 NIKS, with the absence of 16L1 expression possibly reflecting the lower base levels of genome amplification seen with HPV16 (Fig 2A). No obvious differences were noticed however when patterns of differentiation-dependent transcription were compared [13]. We suspect in fact that the different contributions of E4 to the life cycle of HPV16 and HPV18 may reflect differences in the ability of each HPV type to complete their life cycle in the common ‘generic’ keratinocyte background used here, and maybe also to differences in the *in vivo* disease-associations of these two HPV types.

### G2 arrest function of HPV16 E4 contributes to viral genome amplification and L1 expression

**Fig 4:**
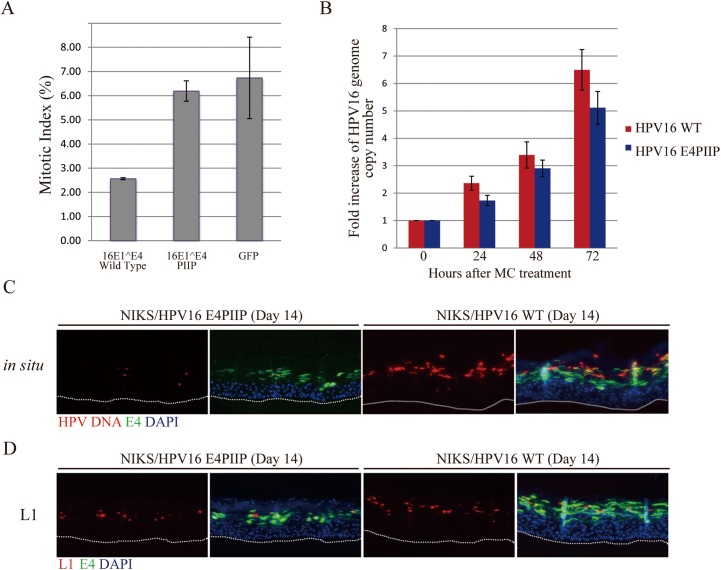
The G2 arrest function of HPV16 E1^E4 contributes to viral genome amplification and L1 expression. **(A)** Mitotic index of Cos-7 cells expressing either the HPV16 WT E1^E4 protein, the E1^E4 PIIP mutant that is defective in G2 arrest function, or a control protein (GFP) that does not inhibit cell cycle progression. The E1^E4 PIIP mutant has lost its ability to prevent mitotic entry when compared to WT 16 E1^E4. **(B)** HPV16 WT or PIIP mutant genome-containing NIKS were suspended in 1.5% methylcellulose (MC) in order to induce differentiation, and were harvested at 0h, 24h, 48h and 72h. Genomic DNA copy numbers were measured by qPCR. The results are presented as fold-increase in HPV16 genome copy number relative to the zero hour time point. A reduction in genome amplification success was noticeable at all time points. **(C & D)** Viral genome amplification and L1 expression were examined at day 14 post-differentiation by fluorescence *in situ* hybridization (FISH signal shown in red in C) and indirect immunofluorescence (L1 staining shown in red in D) in raft tissues derived from NIKS cells harboring either the HPV16 WT or E4KO genomes. Viral genome amplification and L1 expression were compromised in the PIIP mutant. The dotted lines indicate the position of the basal layer. Images were captured using a 10x objective.

Since loss of E4 affected HPV16 genome amplification and L1 expression more dramatically than HPV18, we focused our subsequent analysis on HPV16, and in particular, the role of E4 in this process. A well-established function of 16E4 when expressed at high-level, is its ability to cause cell cycle arrest in a G2-like phase, where viral DNA replication may proceed in the absence of cellular replication [14, 15, 45]. It has been hypothesised that this may facilitate the timely onset of viral genome amplification during epithelial differentiation by antagonizing the proliferative functions of E6 and E7 [45]. Given the clear effect of E4 loss on HPV16 genome amplification, we were prompted to further explore this hypothesis. Our previous studies have shown that HPV16 E1^E4-mediated G2 arrest depends on a PTTP motif at amino acid position 21–24. A mutant 16E1^E4 in which these two threonines were mutated to alanines (i.e. T22A, T23A (PTTP motif changed to PAAP)) no longer inhibited mitotic entry [15]. Since the E4 ORF lies entirely within the E2 gene, the preparation of a G2 arrest-mutant in the context of the full length HPV16 genome could only be made by mutating the E4 PTTP motif to PIIP (E1^E4 T22I, T23I), a modification that introduces only silent changes into the E2 coding sequence. To confirm that the 16E4 PIIP mutation (i.e. T22I, T23I in 16E1^E4) abolishes the G2-arrest function of E4, WT E4 and the 16E1^E4 PIIP mutant were first expressed in Cos-7 cells as described previously [15]. 24 hours after transfection, cells were treated with nocodazole to disrupt mitotic spindle formation, before being left for an additional 24 hours to allow cell-cycle progression. Any cells passing from G2 into M, that are not arrested by E4, will subsequently be arrested in mitosis as a result of the nocodazole treatment. These cells were subsequently visualized by double staining using an anti-16E1^E4 antibody conjugated to Alexa-fluor 488 in conjunction with an antibody to the mitotic marker, phospho-histone H3 and visualization by microscopy. As shown in Fig 4A, the 16E1^E4 PIIP (T22I, T23I) mutant protein no longer inhibited cell cycle progression in G2, with the number of cells passing from G2 to M being broadly similar to what is seen with the GFP control. WT E4 by contrast, reduced the number of cells progressing from G2 to M, which has been shown previously to result from its G2-arrest function. We next prepared the E4 PIIP mutant in the context of the HPV16 genome, before introducing both the WT and mutant genomes into NIKS cells. Both WT and the E4 PIIP mutant genomes were maintained at similar copy number prior to differentiation. To establish how loss of E4’s G2-arrest function affected HPV16 genome amplification, we used the organotypic raft culture system described above, in parallel with a methylcellulose-based differentiation system. Although differentiation in methylcellulose does not perfectly mimic epithelial differentiation *in vivo*, the approach does facilitate quantitation of viral genome copy number. In our experiments, NIKS cells harbouring either the WT or the E4 PIIP genomic mutant were suspended in 1.5% methylcellulose [46], and harvested at 0h, 24h, 48h and 72h. Viral DNA copy number was measured by qPCR, and normalized to cell number using primers directed to the cellular GAPDH gene (see materials and methods). The qPCR results showed that HPV16 DNA copy numbers per cell were slightly reduced in the E4 PIIP genomic mutant, as compared to the WT HPV16 genome at all time-points (Fig 4B), with the trend suggesting statistical significance (P<0.05). To examine the role of the G2 arrest motif more thoroughly, the NIKS cell populations harbouring either the WT or E4 PIIP HPV16 genomes were grown in organotypic raft culture. By *in situ* hybridization and immunofluorescence, less genome amplification and L1 expression were apparent with the 16E4 PIIP mutant genome tissues (Fig 4C and 4D). The finding that L1 levels were reduced but not absent contrasts sharply with the complete loss of capsid protein expression seen with the 16E4 KO genome (see Figs 3B and 4D). As seen for HPV16 E4KO, the E4PIIP mutant genome did not show any obvious differences in the relative abundance of transcripts spanning the early region (S2 Fig), with the reduction in late transcripts being expected because of the lower levels of genome amplification. When taken together, the organotypic raft results appear in broad agreement with the data obtained from the methylcellulose experiments, and indicate that the G2 arrest function of 16E4 contributes importantly to successful life cycle completion. Interestingly, previous work with HPV18 has suggested that the 18E4 G2 arrest function does not contribute in a similar way to the life cycle of HPV18 [47]. We suspect that this may be linked to differences in patterns of E6/E7 expression between the HPV16 and HPV18 rafts, with HPV18 producing a robust and well-ordered productive life cycle in organotypic raft systems. Our previous work has shown that HPV16 produces a range of phenotypes using this model, including those that resemble the CIN 1 and 2 seen in patients [23]. In this setting, it is perhaps not surprising that the anti-proliferative effects of E4 may contribute positively to genome amplification success.

### Different contributions of 16 and 18 E4 to p38 and pERK1/2 MAPK activation during the HPV life cycle

**Fig 5:**
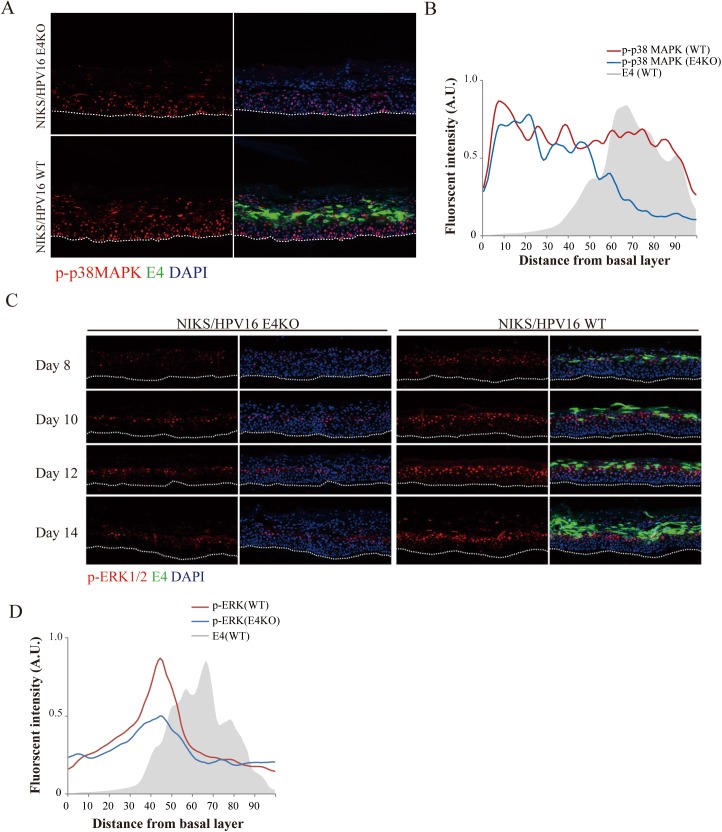
16 E1^E4 contributes to p38 MAPK and ERK1/2 activity during the HPV16 life cycle. **(A)** Raft tissues from NIKS containing HPV16 WT or E4KO genomes were harvested at day 14 post-differentiation and stained for 16E1^E4 (green), phospho-p38 MAPK (p-p38 MAPK) (red) and DNA (blue; DAPI). An elevation of p-p38 MAPK staining in the upper layers of the raft is apparent in rafts generated using the WT HPV16 genome. The dotted lines indicate the position of the basal layer. Images were captured using a 10x objective. **(B)** The extent and intensity of p-p38MAPK staining in the HPV16 WT and E4KO raft tissues at the 14 day time-point post differentiation was digitally scanned from the basal layer to the top of the raft tissue as described in the materials and methods. The expression of E4 in the WT raft is shown as the grey shadow. A very different level of p-p38 MAPK activity was apparent in the upper epithelial layers of rafts prepared using the WT HPV16 genome. **(C)** Raft sections from HPV16 WT or E4KO harvested at 8, 10, 12 or 14 days respectively post-differentiation were stained for 16E1^E4 (green), p-ERK1/2 (red) and DNA (blue; DAPI), to reveal differences in the levels of ERK1/2 activity in the mid epithelial layers. The dotted lines indicate the position of the basal layer. Images were captured using a 10x objective. **(D)** The extent and intensity of p-ERK1/2 staining in NIKS rafts harboring HPV16 WT or E4KO raft tissues at day 14 was examined by digitally scanning the raft tissue from the basal layer to the top of the raft as described in materials and methods. The distribution and intensity of E1^E4 staining in the WT raft is shown as a grey shadow. Comparison with data shown in **(B)** reveals 16E1^E4’s effect on ERK1/2 to be largely confined to the mid epithelial layers, which is distinct from the E4-mediated effect on p38 MAPK which is prominent in the upper epithelial layers.

MAPK (mitogen-activated protein kinase) is an important regulator of viral protein function and viral genome amplification [48], which occurs in the upper layers of HPV-infected epithelial tissue as cells are driven through S-phase and into G2. In addition, it has also been suggested that 16E4 may stimulate p38 MAPK as part of a cellular stress response [49], prompting us to examine whether this additional E4 function may also play a role in HPV genome amplification. To examine this, we first examined HPV16 WT and E4KO day 14 rafts by immunostaining and ImageJ analysis (Fig 5A and 5B). Although p-p38MAPK could be detected in the basal and parabasal layers in both WT and E4KO rafts, the WT rafts showed a marked elevated p-p38 MAPK in the middle and upper layers where E4 accumulation was seen (Fig 5B). Previous studies using undifferentiated monolayer cell culture systems have shown that both E4 and E5 can activate p38 MAPK [49, 50], which is compatible with our observations (S4 Fig). Interestingly, loss of 16E4’s keratin-binding motif (Δ16N), as occurs in the upper epithelial layers where p38 MAPK activation was observed (Fig 5A), did not significantly compromise E4s ability to activate p38 MAPK in this system (S4 Fig), although as expected, N-terminal deletion led to E4 accumulation because of its increased ability to assemble into amyloid-like fibrils [19, 51]. In addition to p38 MAPK [49], HPV16 E4 can stimulate other members of the MAPK family in monolayer cell culture [18], with ERK1/2 activity triggering the initial association of 16E4 with the cellular cytokeratin network [10]. Interestingly, p-ERK1/2 had a similar distribution in both the WT and KO HPV16 rafts, and was confined to the mid epithelial layers close to the point where E4 levels start to accumulate (Fig 5C and 5D). A difference in the intensity of p-ERK1/2 staining was however apparent, with a generally lower signal in the HPV16 E4KO rafts (Fig 5D), with the profiles revealing a different distribution of p-ERK1/2 and p-p38 MAPK in the mid and upper epithelial layers (Fig 5B and 5D). For HPV18, this prominent difference was less apparent when p-p38 levels were compared (S5A and S5B Fig). No apparent difference was seen in either the abundance or the distribution of p-ERK1/2 between HPV18 WT and E4KO rafts (S5C and S5D Fig).

### 16E4 but not 18E4 maintains active cytoplasmic p-JNK MAPK during the HPV16 life cycle

**Fig 6:**
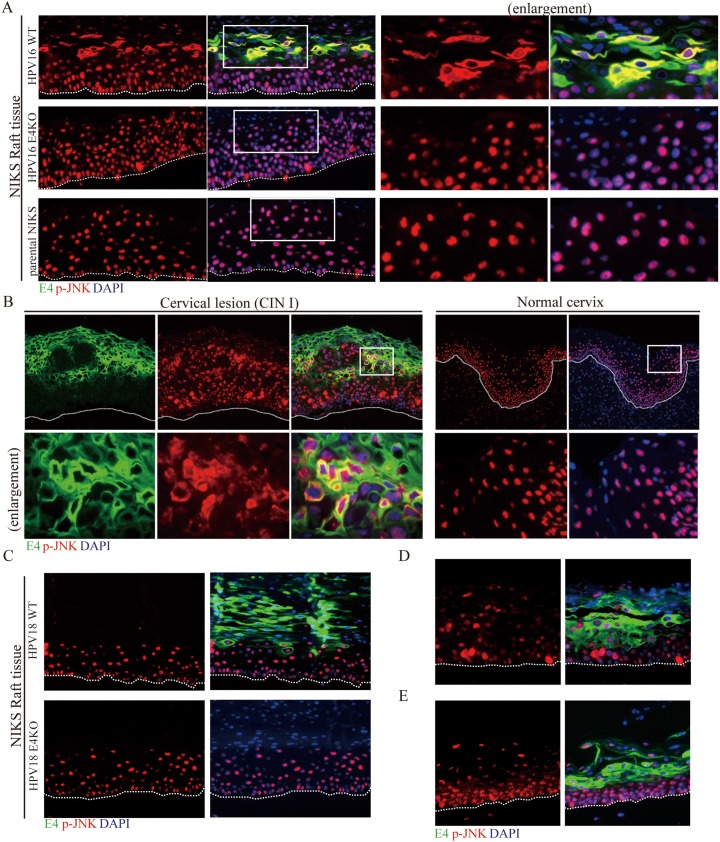
E1^E4 expression is required for the maintenance of activated pJNK in the cytoplasm during the late stages of the HPV16 life cycle. **(A)** Raft tissues derived from NIKS cells or NIKS cells harboring HPV16 WT or E4KO genomes were analyzed at day 14 post-differentiation after staining for 16E1^E4 (green), phospho-JNK (p-JNK, red) and DNA (blue; DAPI). Although elevated cytoplasmic JNK MAPK was apparent before 16E1^E4 became abundant, its activity was noticeably elevated and persisted in cells expressing 16E4. Cytoplasmic p-JNK was not apparent in rafts prepared using ‘empty’ NIKS cells. The boxed areas are enlarged in the right panel. The dotted lines indicate the position of the basal layer. Images were captured using a 10x objective. **(B)** Tissue sections from HPV16-induced cervical lesions or normal cervix were stained for 16E1^E4 (green), p-JNK (red) and DNA (blue; DAPI). The pattern of p-JNK seen in HPV16-induced cervical lesions and in normal cervical tissue was broadly similar to that seen in NIKS-HPV16 rafts and ‘empty’ NIKS rafts respectively. The boxed areas are enlarged in the lower panel. The dotted lines indicate the position of the basal layer. Images were captured using a 10x objective. **(C)** Raft tissues derived from NIKS cells or NIKS cells harbouring the HPV18 WT or E4KO genomes were analysed at day 14 post-differentiation. In contrast to the situation seen with HPV16 (see **(A)** above), no obvious E4-mediated cytoplasmic p-JNK association was apparent. **(D, E)** p-JNK staining in rafts produced from NIKS cells containing either HPV45 (D) or HPV31 (E) show an absence of E4-mediated p-JNK sequestration.

Several studies have implicated elevated MAPK activity and transition into the G2 phase of the cell cycle as being important for HPV genome amplification, and it is notable from the above, that the activity of one member of the MAPK family (i.e. p-ERK1/2), rises transiently in both the HPV16 and 18 rafts in the mid epithelial layers where genome amplification occurs. The HPV E1 protein, which is a DNA helicase/ATPase required for viral genome amplification, is a target for both p-ERK1/2 and p-JNK MAPK as well as CDK1 (a kinase activated in G2), with phosphorylation of E1 affecting its nuclear/cytoplasmic localization [48]. The reduced viral genome amplification seen in 16E4 KO mutants, and the apparent dependency on E4 for HPV16-mediated MAPK elevation, prompted us to extend our analysis of E4s effects to include p-JNK. To our surprise, p-JNK showed by far the most dramatic change in the presence of 16E4, with p-JNK and E4 co-localizing precisely in the cytoplasm of cells in the mid epithelial layers and above (Fig 6A). Cytoplasmic p-JNK was however seen sporadically prior to the onset of E4 expression in the middle layers of the epithelium, but was only ever sustained from the mid to upper epithelial layers when 16E4 was expressed (compare HPV16 E4KO and WT panels in Fig 6A). This sporadic expression of cytoplasmic p-JNK in the basal and parabasal layers prior to its accumulation in E4-positive cells was a direct consequence of HPV presence in the cell, and was never seen in the NIKS parental rafts where the p-JNK patterns were almost exclusively nuclear (Fig 6A, bottom panels). Because the p-JNK/E4 co-localisation pattern was so striking, we next went on to establish whether a similar pattern could be seen *in vivo* in low-grade cervical neoplasia caused by HPV16. A noticeable similarity was apparent when p-JNK and E4 staining in CIN1 was compared to the WT HPV16 rafts, and similarly when uninfected cervix was compared to uninfected NIKS rafts (Fig 6B, left panel). In both instances, 16E4 and p-JNK localize closely as E4 accumulation first begins in the mid epithelial layers, with staining eventually declining as the cells reach the epithelial surface. Although the Cytoplasmic p-JNK staining was more prominent, in both the 16 WT rafts and the HPV16 CIN1 lesion, p-JNK was also apparent in the nucleus of the E4-positive cells (see Fig 6A). Cytoplasmic activated JNK was never observed in normal uninfected cervix (Fig 6B, right panel), which showed a distribution similar to that seen in the NIKS-only rafts. Interestingly, the dramatic E4/p-JNK co-localization seen with HPV16, was not seen in organotypic rafts generated using HPV18 WT or E4KO genomes (Fig 6C), and in this context, it is notable that the other MAPK members were also not notably affected by 18E4 (S5 Fig). Although examined extensively in repeat experiments, the prominent effects seen with HPV16, did not extend to either HPV45 or HPV31 (Fig 6D and 6E), although HPV18, 31 and 45 all showed some evidence of nuclear p-JNK, which tended to decline as E4 levels increased (Fig 6C, 6D and 6E). These results suggest that cytoplasmic pJNK-sequestration during the papillomavirus life cycle may be a HPV16-specific function, rather than a general characteristic of high-risk HPV types. As discussed above, the pJNK pattern seen in HPV16 NIKS rafts was broadly comparable to what was seen in HPV16-associated CIN despite the mucosal epithelial origin of the latter (Fig 6A and 6B).

### HPV16 E4-mediated kinase activation and the modulation of HPV replication proteins represents a novel mechanism by which 16E4 optimises viral genome amplification success

**Fig 7:**
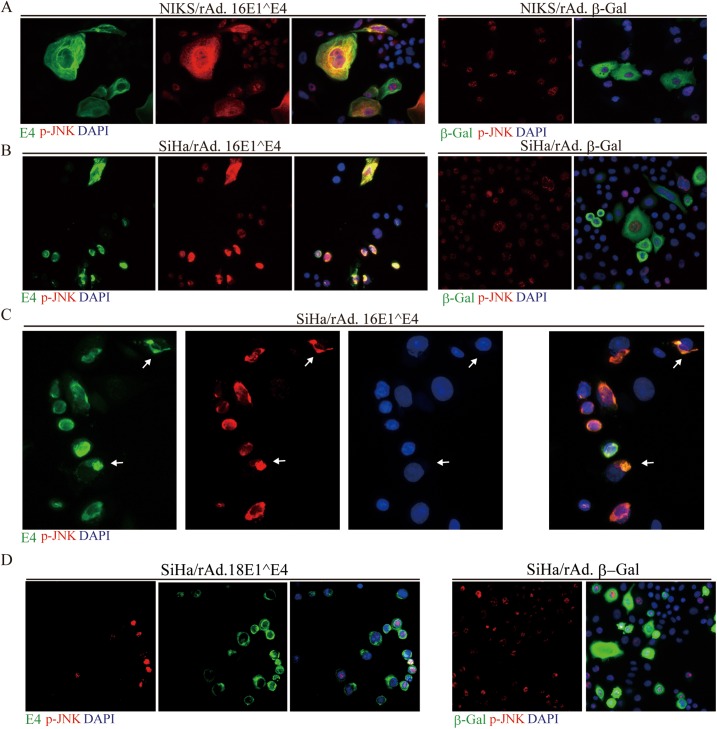
16E1^E4 expression sustains JNK activation and cytoplasmic sequestration. **(A)** NIKS cells were infected for 48 hours with rAd.16E1^E4 or rAd.β-Gal as a negative control. The cells were stained for 16E1^E4 (green) or β-Gal, p-JNK (red) and DNA (blue; DAPI). As seen *in vivo*, 16E1^E4 expression is associated with a dramatic elevation in cytoplasmic p-JNK staining. **(B & C)** SiHa cells were infected for 48 hours with rAd.16E1^E4 or rAd.β-Gal (negative control), before being stained for 16E1^E4 or β-Gal (green), along with p-JNK (red) and DNA (blue; DAPI). In SiHa cells, the p-JNK staining is associated with the perinuclear collapsed E1^E4 keratin networks which can be visualised more clearly in the enlarged image shown in (C). Perinuclear E4/Keratin bundles are arrowed. **(D)** SiHa cells were infected for 48 hours with rAd.18E1^E4 as described above. Cytoplasmic p-JNK staining (red) was not observed. Cells were co-stained for the 18E1^E4 protein (green) and DAPI (blue).

Although use of the organotypic raft culture system, when combined with the analysis of clinically-derived biopsy material remains central to our understanding of HPV biology, the use of undifferentiated cells in cultured monolayer can be used to provide valuable additional mechanistic information. To examine the different abilities of the HPV16 and 18 E4 proteins to modulate p-JNK activity and to consider the consequence of this, the E1^E4 proteins of these viruses were first examined in isolation using undifferentiated epithelial cells grown in monolayer culture. As seen in the organotypic raft system, the HPV16 E1^E4 protein (Fig 7A, 7B and 7C), but not that of HPV18 (Fig 7D) lead to the cytoplasmic accumulation of p-JNK in SiHa cells (Fig 7B and 7C), as well as producing this phenotype in monolayer NIKS cells (Fig 7A). To do this, cells were infected with the recombinant adenovirus expression vectors, Ad.16E1^E4, Ad.18E1^E4 or Ad.βGal, as described previously [15] with E4, β–gal and p-JNK being localized by indirect immunofluorescence as described in the Materials and Methods [49]. These results suggest that 16 E1^E4, but not 18 E1^E4, can sequester active p-JNK in the cytoplasm in absence of other viral proteins, as seen in organotypic rafts. It has previously been shown that 16 E1^E4 associates with the cellular cytokeratin network in both NIKS and SiHa cells, and in SiHa cells promotes network collapse to the nuclear periphery [52]. The characteristic perinuclear E4 distribution is apparent in the enlarged image shown in Fig 7C, and although there is some variation in the p-JNK levels between cells, the co-localisation with 16E4 is reproducibly seen, with some cells showing the characteristic E4/keratin perinuclear bundles (arrowed) (Fig 7C, and [49, 53]).

**Fig 8:**
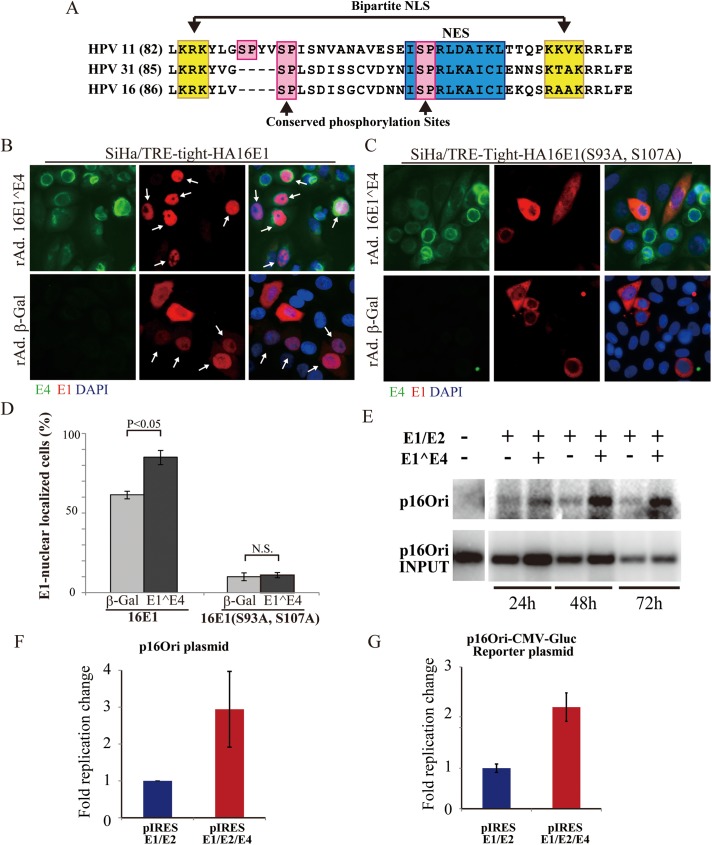
16E1^E4 expression drives nuclear accumulation of wild type but not mutant 16E1 and enhances E1/E2-mediated HPV16 ori-dependent replication. **(A)** Sequence alignment showing the location of conserved MAPK phosphorylation sites involved in regulating E1 nuclear/cytoplasmic shuttling in HPV11, 31 and 16. Numbers in parentheses indicate the position of the first amino acid in each sequence. The location of the bipartite nuclear localization signal (NLS) is indicated by a double-arrow and highlighted in yellow. The nuclear export signal (NES) is shaded in blue. Aligned MAPK- and CDK-phosphorylation sites [S/T]-P, are colored in pink. **(B, C)** SiHa-tetON/CSII-TRE-tight-HA16E1 or CSII-TRE-tight-HA16E1(S93A, S107A cells were infected for 48 hours with rAd16E1^E4 or rAdβ-Gal in the presence of 1 μg/ml of Doxycycline. Cells were stained for 16E1^E4 (green), 16E1 (red; HA-tag) and DNA (blue; DAPI) to show the increased nuclear distribution of wild type E1 but not the phosphorylation-deficient E1 mutant (S93A, S107A) following co-expression with E4. **(D)** The number of cells showing E1-nuclear localization was quantified, and is shown as a percentage of the total number of E1-positive cells counted amongst the E4-positive and E4-negative cells. Wild type E1, but not the phosphorylation-deficient mutant, was enhanced in the presence of E4. The standard deviations of three independent sets of 1000 E1-positive cells are indicated by the error bars. **(E)** To assess replication competence, plasmids expressing 16E1/E2/E4 or 16E1/E2 were transfected along with a HPV 16 replication origin-containing plasmid (p16Ori) as described in the Materials and Methods (S6 Fig). Amplified p16Ori plasmid was analysed by Southern blotting using extracted total DNA (upper panel). The levels of p16Ori input are shown in the lower panel. **(F)** Columns show quantitation of the 72h Southern blot signal averaged across triplicate experiments. Data is shown as ‘fold’ change when compared to the level of replication seen in the absence of E4. **(G)** As in **(F)** above, columns show the change in replication seen in the presence or absence of E4, but in this case, a luciferase reporter plasmid system was used (see materials and methods and S6 Fig). Results were generated from the average of six independent experiments.

The various effects seen with 16E1^E4 on members of the MAPK family, coupled with the ability of 16E1^E4 to arrest cells in G2, suggests that 16E4 may be contributing to genome amplification-success by modulating the intracellular environment to favour the phosphorylation of viral and cellular proteins by these kinases. Although such effects on the cell are likely to be complex and to involve multiple targets, we decided to make a first investigation of this by examining whether E4 expression had any effect on the location of the HPV16 E1 helicase, which is required for genome amplification, and which has been reported to shuttle between the nucleus and the cytoplasm depending on the extent of phosphorylation by members of the MAPK and Cyclin-Dependent Kinase (CDK) families. Amino acid sequence alignment revealed that these phosphorylation sites are conserved amongst the E1 proteins of HPV16, HPV31 and HPV11, with the latter containing an additional upstream phosphorylation site (see Fig 8A and [48, 54, 55]). Our initial E1/E1^E4 co-transfection experiments resulted in only a small number of cells co-expressing the two proteins. To overcome this difficulty, we established an inducible cell line in which a HA-tagged 16E1 protein is expressed from a doxycycline inducible promoter using a lentivirus CSII-TRE-tight-HA16E1 construct. A SiHa background was used for these experiments, as SiHa cells constitutively express the HPV16 E6 and E7 proteins, that would be expressed along with E1^E4 and E1 in cells supporting viral genome amplification *in vivo*. We were unable to generate cell lines constitutively expressing the E1 protein however, presumably because of E1-mediated cell toxicity following overexpression. Lentivirus infection was carried out into Tet-On SiHa cells prepared using retrovirus pQCXIzeo-tetON ADV followed by selection in zeocin as described in the Materials and Methods. In agreement with the hypothesis that E4 expression may modulate the function of other viral gene products, a very clear increase in E1 nuclear localization was apparent when expression was carried out along with E4 in the same cell (Fig 8B), with E4 expression leading to a statistically significant change in intracellular E1 distribution (Fig 8D). Statistical analysis was facilitated by combining the inducible E1 expression system with use of the rAd16E1^E4 expression vector, which ensured E1/E4 double-positivity in the majority of cells expressing E1 in the dish. In contrast to the wt 16E1 protein, the phospholyation site-deficient E1 protein (E1S93A.S107A) was found primarily in the cytoplasm, with the presence or absence of 16E4 having little effect on its intracellular location (Fig 8B and 8C). The % of cells showing nuclear E1 in the presence or absence of E4 was calculated from the analysis of 3000 E1-positive cells is shown in Fig 8D.

Given that the presence of E4 can facilitate E1 nuclear accumulation, we next wanted to establish whether E4 could also enhance E1/E2-mediated viral genome replication in a functional assay. Because E1, E2 and E4 are not expressed at equivalent levels during genome amplification, a HPV16 viral DNA fragment encoding just the E1/E2/E4 region was first isolated from the HPV16 WT or E4KO genome, and cloned downstream of a CMV promoter positioned immediately in front of E1 (S6A Fig). With respect to the E1, E2 and E4 open reading frames, the position of the CMV promoter in these clones (pIRES-16E1/E2/E4 or pIRES-16E1/E2) mimics that of the HPV16 late (p670) promoter in the viral genome, which drives the co-ordinated expression of E1, E2 and E1^E4 during viral genome amplification. In agreement with data shown in S2 Fig, the pIRES plasmids exhibited similar transcription profiles across the E1, E2 and E1^E4 regions to those seen with the full-length viral genome (S6B Fig), with E2 and E4 also being detectable by western blotting (S6C Fig). As expected, E4 was not apparent in cells transfected with the E4KO plasmid, (pIRES-16E1/E2), and in the absence of a HA tag, E1 protein could not be reliably visualized using available antibodies (S6C Fig). In fact E1 transcript levels were tightly regulated in this system, just as they are during the HPV16 life cycle [56–60]. Interestingly, and as reported previously, total E2 levels were always elevated in the presence of E4, in agreement with previous studies showing that E2 can become sequestered and stabilized in the cytoplasm in undifferentiated cells expressing both proteins together [61]. In addition to E1 nuclear localization therefore, it remains a possibility that E2 cellular accumulation may also contribute to E4’s overall effects on genome amplification. The precise mechanism by which E4 affects E2 abundance, and the possible effects of MAPK phosphorylation are not however known, although a direct interaction between E1^E4 and E2 in the cytoplasm has been previously reported [61]. As with most HPV early proteins [62], the detection of E1 has never been shown in raft tissue, and the detection of HPV16 E2 during productive infection has only been convincingly reported with a single purified rabbit polyclonal antiserum [35], with other antibodies to the HPV16 E2 protein failing to detect the protein during productive infection using immunofluorescence or immunohistochemistry protocols. Unfortunately, this purified antibody is no longer available from the researchers who originally produced it (Dr Yuezhen Xue, Institute of Medical Biology, Singapore, personal communication), although we were able to obtain a small aliquot of the unpurified rabbit serum. Curiously, our optimized staining protocol using this antibody did appear to show an elevated in E2 signal in the middle layer of HPV16 WT rafts, when compared with E4KO rafts (S7A and S7B Fig), with nuclear E2 staining apparent at around the time of E4 elevation (S7B Fig). Although background staining was higher than we would have liked using this antibody, the conclusion was supported by digital scanning across the length of the raft tissue (S7C Fig). In contrast to the situation in undifferentiated monolayer cells however, the detectable E2 protein seen in the differentiated raft cultures was predominantly nuclear in our hands, although previous studies have reported both nuclear and cytoplasmic E2 protein using this antibody [63]. In an attempt to substantiate these observations, we next went on to examine E2 by western blotting (S7D, S7E and S7F Fig), using the microdissection approach used previously to assess L1 accumulation. E2 antibody specificity was first assessed against 16E2 expressed from endogenously expressed E2 in 293T cells (S7D Fig), before being examined in organotypic raft extracts. The results were broadly compatible with the immunofluorescence staining, and was reproducibly seen in the replicate rafts prepared here (S7E and S7F Fig). Although we suspect that 16E4 most likely affects E2 function as well as E1, in the absence of more robust E2 detection reagents and a better understanding of E2 MAPK phosphorylation, a contribution to the E1 recruitment to the viral origin of replication may be expected.

Given the above effects of 16E1^E4 on the viral replication proteins, and in particular its effect on E1 nuclear localization, we next examined the contribution of 16E1^E4 on E1/E2-mediated replication using the modified *in vitro* replication assay. In the first instance, pIRES-16E1/E2/E4 and pIRES-16E1/E2 were transfected into monolayer C33a or SiHa cells along with a HPV16 replication origin-containing plasmid (p16Ori [41]). Despite variation in pOri input levels, E4 (pIRES-16E1/E2/E4) appeared to enhance E1/E2-mediated replication efficiency (Fig 8E and 8F). To confirm these results, and to avoid the inaccuracies that can result from the measurement of band intensities (see Fig 8E), a modified reporter plasmid was prepared (p16Ori-CMV-GLuc (S6 Fig)), to allow replication efficiency to be estimated from the Gluc/Cluc ratio after co-transfection with a pCMV-CLuc reporter plasmid. In this system, the presence of 16 E4 reliably enhanced E1/E2-mediated replication success in repeat (6x) experiments (Fig 8G).

**Fig 9:**
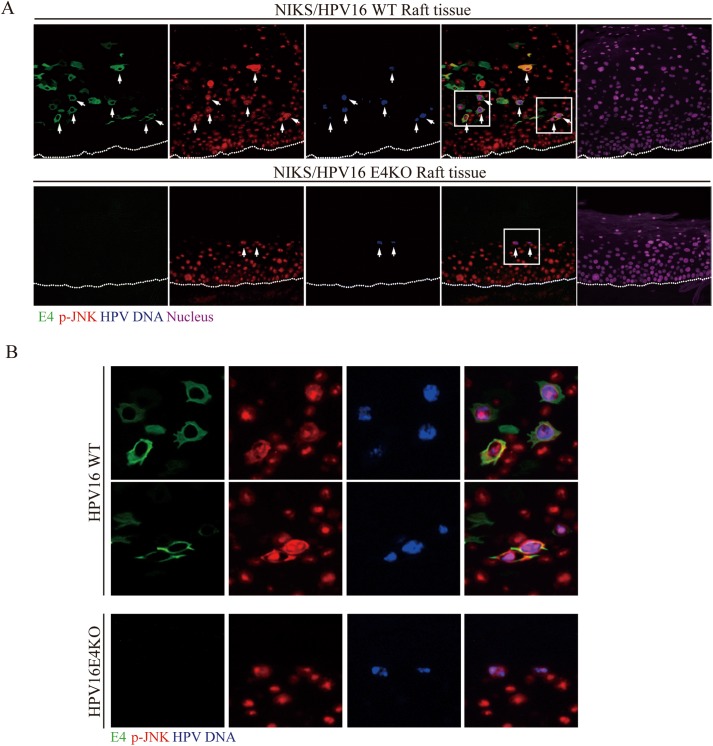
Viral genome amplification during the productive life cycle occurs in cell co-expressing both 16E1^E4 and activated cytoplasmic JNK. **(A)** Raft sections derived from NIKS cells harboring HPV16 WT or E4KO genomes were analysed at day 14 post-differentiation by quadruple staining for E1^E4 (green), p-JNK (red), nuclear DNA (magenta; DAPI, colour modified using imageJ) and HPV16 genomic DNA (blue, *in situ* hybridization, colour modified using imageJ). Efficient viral genome amplification is confined to the staining for cytoplasmic E4 and cytoplasmic phosphor-JNK-positive upper epithelial layers of WT rafts. In the E4KO rafts a lower level of genome amplification can occur in cells that are negative for cytoplasmic phosphor-JNK. Such cells are typically in the upper p-JNK-staining layers and typically show only nuclear p-JNK staining. The dotted lines indicate the position of the basal layer. Images were captured using a 10x objective. **(B)** Enlarged areas of the regions boxed in (A) to show the E4/p-JNK patterns in cells known to be supporting HPV16 genome amplification.

When taken together, the above results suggest a mechanism by which 16E4-mediated MAP kinase modulation facilitates replication success, and also predicts that genome amplification, the activation of MAP kinase, and the accumulation of 16E4 should occur simultaneously during the late stages of the HPV16 life cycle. To establish whether this is indeed the case, a quadruple stain was carried out to localize the sites of viral genome amplification (as determined by fluorescence *in situ* hybridisation (FISH)) with E4, p-JNK and DAPI. In the HPV16 WT rafts, a remarkable co-localisation of all three markers was apparent, with genome amplification occurring only in cells showing positivity for cytoplasmic p-JNK that were also positive for 16E4 (Fig 9, upper panel, arrowed). A much lower level of genome amplification was seen in the E4KO rafts, in the absence of E4 in cells that were always negative for activated cytoplasmic p-JNK (Fig 9, lower panel, arrowed). As expected, and in line with other reports, it was not possible to detect E1 during the productive life cycle with available antibodies [64]. When taken together with our E4 *in vitro* expression studies, this data strongly supports the idea that 16E4 acts to modulate the cellular environment during the late stage of infection via the MAPK activation, and that this influence the activity and/or localisation of viral proteins more directly involved in viral genome amplification.

## Discussion

Conflicting data have been reported as to the role of E4 during the virus life cycle, especially in its contribution to viral genome amplification amongst different HPV types [13, 20–22]. To address this issue, and to examine the mechanistic roles of E4 during productive infection, we have carried out a careful comparative analysis of E4’s role in genome amplification between the two most important high-risk HPV types, HPV16 and 18. To do this, we made use of WT and E4KO genomes maintained in an isogenic keratinocyte cell background in order to eliminate variation between different keratinocyte pools and differentiation protocols. Experiments were carried out using matched cell population harboring WT and E4KO genomes of either HPV16 or 18 at similar copy number and at similar passage. Finally, because the HPV life cycle is critically dependent on the differentiation status of the infected host cell, effects on viral genome amplification were examined using raft culture time course experiments rather than at a single raft time point, with digital imaging being used to help determine the significance of changes in patterns of gene expression. Using these approaches, we conclude that while E4 can contribute to virus replication efficiency and life cycle completion, it is not essential for these events. In the isogenic NIKS background, loss of 16E4 was found to have a much more marked effect on life-cycle-success than 18E4, allowing us to identify a role for 16E4’s G2 arrest function as well as its ability to modulate members of the MAPK family, most notably JNK, which we show to be a prominent modification seen during natural HPV16 infection *in vivo*. The elevation of p-JNK, which was not seen in HPV18, once again highlights differences in the life cycles of high-risk viruses. For HPV16, it appears that E4, and its effects on MAPK affect E1 nuclear localisation, and that in both transient replication assays and during epithelial differentiation, that 16 enhances replication success as a result.

Previous reports have suggested that the loss of full length E4 may enhance the growth rate in undifferentiated HPV18-containing primary foreskin keratinocytes [21], possibly because the over-expression of 18E4 in monolayer culture can cause cell cycle arrest at G2/M. The growth rate of WT or E4KO genome-containing NIKS populations in our system were comparable (Fig 1), which fitted with our observation that E4 is not detectable in undifferentiated monolayer cell culture, either by immunostaining or western blotting, and is unlikely to disrupt cell-cycle progression at G2. By contrast, high-level expression of the E4 protein of several types, including HPV16 and 18 induces cell cycle arrest at G2/M in monolayer-cultured cells [15, 16]. During the HPV life cycle, the expression of the E4 protein is controlled by the HPV late promoter (p670 in HPV16, p811 in HPV18), which is driven by the epithelial differentiation machinery. Although not always apparent, differentiation can occur to some extent in keratinocyte monolayer culture post confluence, which may have contributed to the previous observation [21]. In our hands using NIKS, we find that the presence of HPV16 or 18 episomal genomes is a strong suppressor of differentiation in monolayer culture, with E4 expression not apparent over the time course of our experimental growth assays. Given that we also saw no effect of E4-loss on either epithelial thickness or the extent of cell cycle entry in raft culture, we do not feel that E4 has any obvious role during the early stages of either the HPV16 or 18 life cycles. A similar conclusion was reached when E4 loss was assessed in the CRPV model of infection *in vivo* [44].

Although the loss of E4 delays viral genome amplification in both HPV16 and HPV18, the effect was much more dramatically seen with HPV16. This allowed us to examine specific E4 functions in the context of the HPV16 life cycle, especially in viral genome amplification. In repeat experiments, our stringent analysis showed that the loss of the G2-arrest motif in HPV16 leads to a noticeable and consistent reduction in genome amplification success, suggesting that G2 arrest function of E4 in HPV16 contributes to viral genome amplification efficiency. In HPV-induced lesions or in HPV containing raft tissues, E4 expression starts before E6/E7 levels decline [29], with the expression of these proteins overlapping in the middle epithelial layers where viral genome amplification occurs [7, 29]. The G2 arrest function of E4 is thought to suppress E7-mediated cell proliferation in the upper epithelial layers and to prolong the G2-like state that is required for efficient viral genome amplification (reviewed in [45]). From our results, this hypothesis remains reasonable for HPV16. There is however some uncertainty regarding the role of E4 mediated G2-arrest in the life cycle of HPV18. Disruption of a motif required for G2/M arrest in the E4 ORF of HPV18 genomes did not affect HPV18 genome amplification [47]. Although we have not tested the effect of the G2 arrest function of E4 on genome amplification in HPV18 in our system, in our raft culture time course experiment, a reduction in genome amplification was seen only at early time points with the HPV18 E4KO mutant, and was not apparent at late time points. The expression pattern of E4 is similar between the HPV16 and 18 WT, which may suggest that the G2 arrest function of HPV18 E4 may sometimes contribute to HPV18 genome amplification efficiency as seen here with HPV16. From our previous analysis of HPV gene expression in model system of disease [23] and in clinical material [24, 29], we suspect that this may be apparent at epithelial sites where there is some deregulation of E6/E7 expression rather than in the raft model, which for HPV18 (as well as HPV31 and 45) appears to be a model of well-ordered productive infection rather than neoplasia. Raft culture does not however mimic the precise characteristics found during *in vivo* infection, and we are not yet able to model the different epithelial sites where HPV18 naturally causes disease. The different abilities of HPV16 and HPV18 to complete their life cycle in organotypic rafts does however suggest important differences in tropism, and that a common cell type is unlikely to support the life cycles of different HPV types equally. Indeed, this was an important consideration when deciding to carry out our evaluation of two HPV types in an isogenic cellular background. Interestingly, although we were able to match genomic copy number in the WT and E4KO cell populations and lines, the copy number of established HPV-containing NIKS was always different in cell populations prepared using HPV16 and 18. It is clear from this that HPV16 and 18 have different regulation of maintenance replication in the isogenic NIKS background, again reflecting differences between these two viruses.

It appears however that other functions of E4 can contribute to the enhancement of viral genome amplification efficiency. This study suggests that 16E4’s contribution to viral genome amplification efficiency may also involve the activation of MAPK (mitogen-activated protein kinase). The MAPK super-family is composed of ERKs, JNK/SAPKs and p38 MAPKs [65]. In HPV11 and HPV31, the E1 protein, a DNA helicase/ATPase involved in initiating viral DNA replication, shuttles from the cytoplasm into the nucleus after phosphorylation by ERK and/or JNK, and remains in the nucleus because of the presence of activated CDK1/2, which can phosphorylate a CRM-1 dependent NES which inhibits E1 nuclear export, allowing an enhancement of viral DNA replication with appropriate timing during productive infection (reviewed in [55]). The work presented here reveals that E4 loss in the context of the HPV16 genome results in a significant reduction in sustained ERK, JNK and p38 MAPK activity in the upper layers of the epithelium, with these proteins being absent in genome amplifying cells in the HPV16 E4KO raft tissues. In addition to this, activated JNK was found to be prominently associated with HPV16 E1^E4 in the cytoplasm of cells supporting HPV genome amplification, both in the WT HPV16 rafts, but also in low-grade cervical disease caused by HPV16. In fact the patterns of E4 and JNK expression, and their correlation with vegetative viral genome amplification were very similar in the HPV16 WT rafts and in the patient biopsies, which strengthen both the significance of our observations, and our confidence in the NIKS organotypic raft system. Interestingly, our previous work indicated that HPV16 E1^E4 stimulated JNK and CDK activity when expressed in SiHa cells [14, 49] and retained CDK1/cyclin complex in the cytoplasm and induced G2 arrest. As mentioned above, both ERK and JNK activation are important for E1 phosphorylation, with E1 phosphorylation being important for viral DNA replication. Like HPV11 E1, the HPV16 E1 protein sequence contains a consensus MAPK docking domain that is essential for ERK, JNK and p38 MAPK binding [48, 66], and a consensus cyclin binding motif (RxL). A plausible hypothesis therefore is that HPV16 E1^E4 may activate MAPK and CDKs to modulate E1 nuclear localization in order to facilitate viral genome replication. The observations here, that HPV16 E1^E4 expression facilitates HPV16 E1 nuclear localization and that HPV16 E1 lacking conserved putative phosphorylation sites failed to localize in the nucleus, support the idea that 16E1^E4 can contribute to viral genome amplification by facilitating the unclear localization of E1 as a result of phosphorylation by kinases such as MAPKs and CDKs activation. Interestingly, HPV16 E4 is also a target for self-phosphorylation by activated MAPK, which occurs during viral genome amplification [18], and which enhances the association with cytokeratin and E4 protein stability. This is thought to trigger E4 protein accumulation, and prolong the G2 arrest period to facilitate optimal viral genome amplification. Interestingly, a recent report has suggested that the phosphorylation of E5 by activated p38 MAPK in HPV18 was important for viral genome replication (Tom Broker, University of Birmingham, Alabama, USA; personal communication). Our previous studies have shown that HPV16 E4 activates p38 MAPK directly as a result of an E4 triggered cell stress response [49], and the results shown here, as well as those presented previously, suggest E4 may also augment E5 and E2 function to some extent [61]. Interestingly, 16 E4 has been reported to be able to bind and stabilise E2 in the cytoplasm [61], and just as with E1, the E2 protein can also be regulated by the cellular CDK1/2 kinases, which lead to an increase in E2 stability during S-phase [67]. In addition, E1^E4 and E5 are expressed together from an abundant bicistronic viral transcript during the late stages of the virus life cycle, a pattern of expression that fits well with their possible functional association in stimulating and sustaining the activity of MAPK. Clearly E4 has other roles during the late stages of infection, and our data suggest that N-terminally cleaved E4, which assembles into E4 amyloid-like fibers, may activate p38 MAPK in the later stage of viral life cycle, with a potential role in virus transmission and release (data presented here & [19], [49]). The extended p38 MAPK activation apparent in the upper layers of HPV16 WT raft tissues supports this hypothesis. How E4 is associated with ERK activation is not currently answered. Unlike pJNK, the level of phosphorylated pERK was not changed when HPV16 E4 was expressed in SiHa cells by transfection with a 16E1^E4 expression vector or following rAd.16E1^E4 infection (data not shown). Indeed, ERK activation is thought to be mediated primarily by E7 and E5 [18]. Similarly, the co-expression of E2 and E4 did not alter ERK activation (data not shown), although the E4/E2 association reported previously led to E2 stabilization [61], at least in the SiHa model. For JNK MAPK, we suspect that 16E4 role may be to sequester the kinase onto the abundant E4 amyloid structures, and that in the upper epithelial layers, the role of E6, E7 and E5 are critical for its presence. In this case, some similarity is apparent with the prominent E4-mediated sequestration of CyclinB/Cdk1 seen in lesions caused by Mu PV types such as HPV1, and Lambda papillomaviruses such as the Canine or Rabbit Oral Papillomaviruses [14].

A key reason for initiating this study arose from the need to clarify whether E4 loss abolishes genome amplification, or whether E4 acts to modulate amplification-efficiency but is not essential for genome amplification *per se*. Our data demonstrate that loss of full length E1^E4 expression in HPV18 acts to delay differentiation-dependent viral DNA amplification and L1 expression. These data are slightly different from the previous report, which showed impaired or abolished genome amplification and late gene expression in HPV18 E4KO. These differences may come from the different approaches used, and indeed our raft time course experiments show that both conclusions could be reached depending on the time point analyzed. Indeed, the culture of primary foreskin keratinocytes containing HPV18 WT and HPV18 E4KO genomes using the organotypic raft system, leads to similar results to those shown here as culture times are extended, with both WT and mutant genomes producing similar infectious titres (Craig Meyers, Penn State College of Medicine (personal communication)). Our conclusions were facilitated by carrying out raft culture time course coupled with digital scanning, which can reveal the different degree of epithelial differentiation and the relative changes in protein expression. Poor genome amplification is always more apparent at early stages of differentiation in the E4KO raft tissues, with raft time courses and differences in the time of analysis explaining many of the previous discrepancies. With HPV16, an abolition of genome amplification is apparent at the early time point, whereas with the 18 rafts, it would looks as though E4 has no effect on genome amplification if only the last time point is evaluated. These are the two extremes. By looking at all the time points together we can get the clearest conclusion that E4 acts generally to optimize replication efficiency, but is not essential for viral genome amplification. To some extent, this conclusion may apply to all high risk HPV types, although clearly the contribution of E4 is not uniform, and the sequestration of JNK in particular appears a characteristic of the HPV16 productive cycle. A different result was previously reported during the low risk HPV11 life cycle, which may reflect the very different functions of the low risk E6 and E7 genes, and the different ability of these proteins to drive cell proliferation. Interestingly, the ability of HPV18 to complete its life cycle in the absence of E4 in the NIKS model, will allow us in future to consider the role of E4 in virus assembly, virus maturation, and transmission, where the E4 proteins, including those of HPV16 and 18 almost certainly have their primary function.

When the data is taken together, we conclude that loss of full length E4 in HPV16 and 18 only delays viral genome amplification and L1 expression, but does not abolish these events in the virus life cycle. For HPV16, this delay appears to be mediated by several of E4’s activities including its G2 arrest function, and its role in activating members of the MAPK pathway, such as ERK, JNK and p38, with sustained JNK activation being a HPV16-specific characteristic that affects E1 nuclear localisation, and as a result of this, replication efficiency. Previous studies showing effects of 16E4 on E2 stability and cytoplasmic accumulation [61], and the cross-talk between 16E4 and E5-mediated kinase stabilisation most likely represent additional E4-associated modifications involved in this process. The massive accumulation of E4 in the upper epithelial layers, coupled with its ability to assemble into amyloid-like fibres, must however underlie an additional and perhaps more critical role for E4 post-genome amplification.

## Supporting information

S1 Fig HPV episomal status and genomic copy number in NIKS cell populations and clonal cell lines. **(A)** Southern blot of DNA extracted from three HPV16 clonal cell populations (T1, T2 and T3) after digestion with *Bam* HI (B) and *Hind* III (H). The track labelled (-) contains DNA extracted from the parental NIKS population. *Hind* III digestion allowed visualisation of mostly supercoiled (SC) viral genomic DNA, along with open circle (OC) and linear (L) forms. *Bam* HI, which cuts once in the HPV16 genome, gave rise to a single 8kb linear band (L). *Bam* HI-linearized HPV-16 control genomic DNA (tracks labelled ‘DNA’) was run alongside DNA isolated from the clonal cell populations as a size marker. Slower-migrating genomic DNA visible at higher loading is marked by an asterisk.
**(B)** To confirm that viral gene expression is primarily from episomal HPV, an APOT assay was performed on the cell lines and cell populations used in this study. The clonal cell populations (T1,T2 and T3) shown in (A) express a predominant E6/E7 transcript of approximately 1Kb, whereas cell lines with integrated HPV DNA typically contain heterogeneous transcript patterns comparable to those shown in track labelled ‘IN’. A ‘no RNA’ loading control is shown in track (-).
**(C & D)** HPV copy number-diversity was established in 18 individual HPV16 **(C)** and HPV18 **(D)** clonal cell populations. While all cell lines harbored episomal genomes, the copy number varied between individual clones, presumably reflecting copy number variation in individual cells in the HPV16 and 18 populations. Copy number matched clones and populations were used for the comparative analysis described here.
(TIF)

S2 Fig Transcripts spanning E1, E2, and the E1^E4 splice junction are expressed at similar levels from both WT and E4KO genomes. **(A)** Viral transcripts spanning E1, E2, or using the E1^E4 splice junction (880^3358), were quantified after reverse transcription (RT) by qPCR as described in Materials and Methods. Transcript abundance was normalized against total early transcripts measured using qPCR primers located immediately upstream of the early polyadenylation site and within the E5 ORF (columns labeled E5). In the absence of the RT step, the qPCR procedure produced negligible signal with all primer sets (mean 0.16%; SD 0.18%). No significant differences were apparent between the WT HPV16, the E4KO and E4PIIP genomes, suggesting that the presence of E4 does not affect patterns of transcription.
(B) The ability of the E1^E4 primers to detect only the spliced E1^E4 transcript was assessed against a 10-fold dilution series of cloned E1^E4 cDNA (orange crosses/line) or unspliced HPV16 genomic DNA (blue crosses). The E1^E4 primers were amplified a PCR product only from spliced cDNA.
(TIF)

S3 Fig Organotypic rafts prepared using WT and E4KO genomes are not obviously compromised in their ability to differentiate. **(A)** Rafts prepared using HPV16 WT or E4KO genomes are shown at day 10 and day 14 after staining with Hemotoxylin and Eosin (H&E, upper panels). The middle panels show immunofluorescence stains for E4 (green) and keratin 10 (K10, red), with the lower panels showing staining for E4 (green) and filaggrin (red). Immunofluorescence images are counterstained with DAPI (blue) to allow visualization of the cell nuclei.
**(B)** Rafts prepared using HPV18 WT or E4KO genomes and stained with H&E, or to establish the patterns of K10 and filaggrin expression as described above.
(TIF)

S4 Fig p38 MAPK phosphorylation in the presence or absence of 16E4 or 16E4ΔN. **(A)** 16E1^E4 was expressed from rAd16E1^E4 (tracks labelled E4+) in SiHa and SiHa_E5 cells (tracks labelled E5+). SiHa_E5 cells have been described previously [18]. Levels of activated p38 are shown in track labelled p-p38. The effects of 16E1^E4 on pERK1/2 in this system have been described previously [18].
**(B)** The 16 E1^E4 protein or the N-terminally deleted form of 16 E1^E4 were expressed in SiHa cells as described in Materials and Methods. Levels of activated p38 are shown in track labelled p-p38.
(TIF)

S5 Fig HPV 18E4 does not significantly contribute to p38 MAPK and ERK1/2 activity during the HPV18 life cycle. **(A)** Raft tissues from NIKS containing HPV18 WT or E4KO genomes were harvested at day 14 post-differentiation and stained for 18E1^E4 (green), phospho-p38 MAPK (p-p38 MAPK) (red) and DNA (blue; DAPI). A modest elevation of p-p38 MAPK staining in the upper layers of the raft is apparent in rafts generated using the WT and E4KO HPV18 genome with no significant differences between the two genomes. The dotted lines indicate the position of the basal layer. Images were captured using a 10x objective.
**(B)** The extent and intensity of p-p38MAPK staining in the HPV18 WT and E4KO raft tissues at the 14 day time-point post differentiation was digitally scanned from the basal layer to the top of the raft tissue as described in the materials and methods. The expression of E4 in the WT raft is shown as the grey shadow. A similar level of p-p38 MAPK activity was apparent in the upper epithelial layers of rafts prepared using the WT and E4KO HPV18 genome.
**(C)** Raft sections from HPV18 WT or E4KO genomes were harvested at 14 days post-differentiation and stained for 18E1^E4 (green), p-ERK1/2 (red) and DNA (blue; DAPI), to reveal differences in the levels of ERK1/2 activity in the mid epithelial layers. The dotted lines indicate the position of the basal layer. Images were captured using a 10x objective.
**(D)** The extent and intensity of p-ERK1/2 staining in NIKS rafts harboring HPV18 WT or E4KO raft tissues at day 14 was examined by digitally scanning the raft tissue from the basal layer to the top of the raft. The distribution and intensity of E1^E4 staining in the WT raft is shown as a grey shadow. In contrast to HPV16, 18E1^E4 had little effect on ERK1/2, which was largely confined to the mid epithelial layers.
(TIF)

S6 Fig pIRES-16E1/E2/E4 and pIRES-16E1/E2 gene expression plasmids. **(A)** Schematic representation of the E1/E2/E4 and the E1/E2 expression vectors (pIRES-16E1/E2/E4 and pIRES-16E1/E2). DNA fragment containing intact E1, E2 and E4 genes, or the E1 and E2 genes along with the E4KO gene, were cloned downstream of the CMV promoter in pIRESeGFP. The E4 ORF is contained within the E2 ORF but is translated in different reading frame from a sliced mRNA. The GFP protein is translated from the same transcripts using an internal ribosome entry sites (IRES).
**(B)** Viral transcripts spanning E1, E2, or using the E1^E4 splice junction (880^3358), were quantified after reverse transcription (RT) by qPCR as described in Materials and Methods. As shown above, the ratio of transcripts spanning E1 or E2, or using the E1^E4 splice junction, were similar to that seen from the HPV16 WT or E4KO genomes (S2 Fig).
**(C)** C33a cells were transfected with pIRES-16E1/E2/E4 or pIRES-16E1/E2. Cell extracts were analyzed by Western blotting with antibodies to E2, E4, tublin and GFP. In the absence of good E1 antibodies, E1 expression was visualized by RT-PCR as described in **(B)** above.
**(D)** The structure of the p16 Origin plasmid (p16Ori) and the p16Ori reporter plasmids (GLuc and CLuc) are shown diagrammatically. These plasmids were used in the replication assays shown in Fig 8.
(TIF)

S7 Fig E2 is stabilized in the presence of E4. **(A)** Detection of E2 in HPV16 WT and E4KO rafts and correlation with E4 expression. E2 staining was carried out using the rabbit polyclonal antibody described in [35] without purification. Although some background staining was apparent with this antibody, nuclear staining, which overlapped the DAPI signal, was seen in cells positive for E4 (arrowed). Such staining was always less distinct in the 16 E4KO raft.
**(B)** Enlarged image of cells arrowed to show the E2 (red) and E4 (green) immunofluorescence staining in the mid epithelial layers of the 16E4WT raft.
**(C)** Digital imaging revealed a lower overall E2 signal, particularly in the mid-epithelial layers, when 16E4WT and E4KO rafts were compared.
**(D)** Total protein extracted from 293T cells transfected with either empty or HPV16 E2 expression vector are shown in tracks labelled (-) and E2, following Western blotting using the E2 rabbit polyclonal antisera described in [35].
**(E)** Total protein was extracted from microdissected raft epithelium (WT or E4KO) at the 14 day time point, and the levels of E2 protein visualised by Western blotting as in (**D**).
**(F)** Columns show quantitation of the Western blot signal averaged across triplicate experiments and normalized to GAPDH. Data is shown as ‘fold’ change when compared to the E2 levels observed in the presence of E4.
(TIF)
