# Supplementary figures and images for: HPV16 and 18 genome amplification show different E4-dependence, with 16E4 enhancing E1 nuclear accumulation and replicative efficiency via its cell cycle arrest and kinase activation functions
Source: PLoS Pathog. 2017 Mar 17;13(3):e1006282. doi: 10.1371/journal.ppat.1006282 (PMC5371391; doi:10.1371/journal.ppat.1006282)

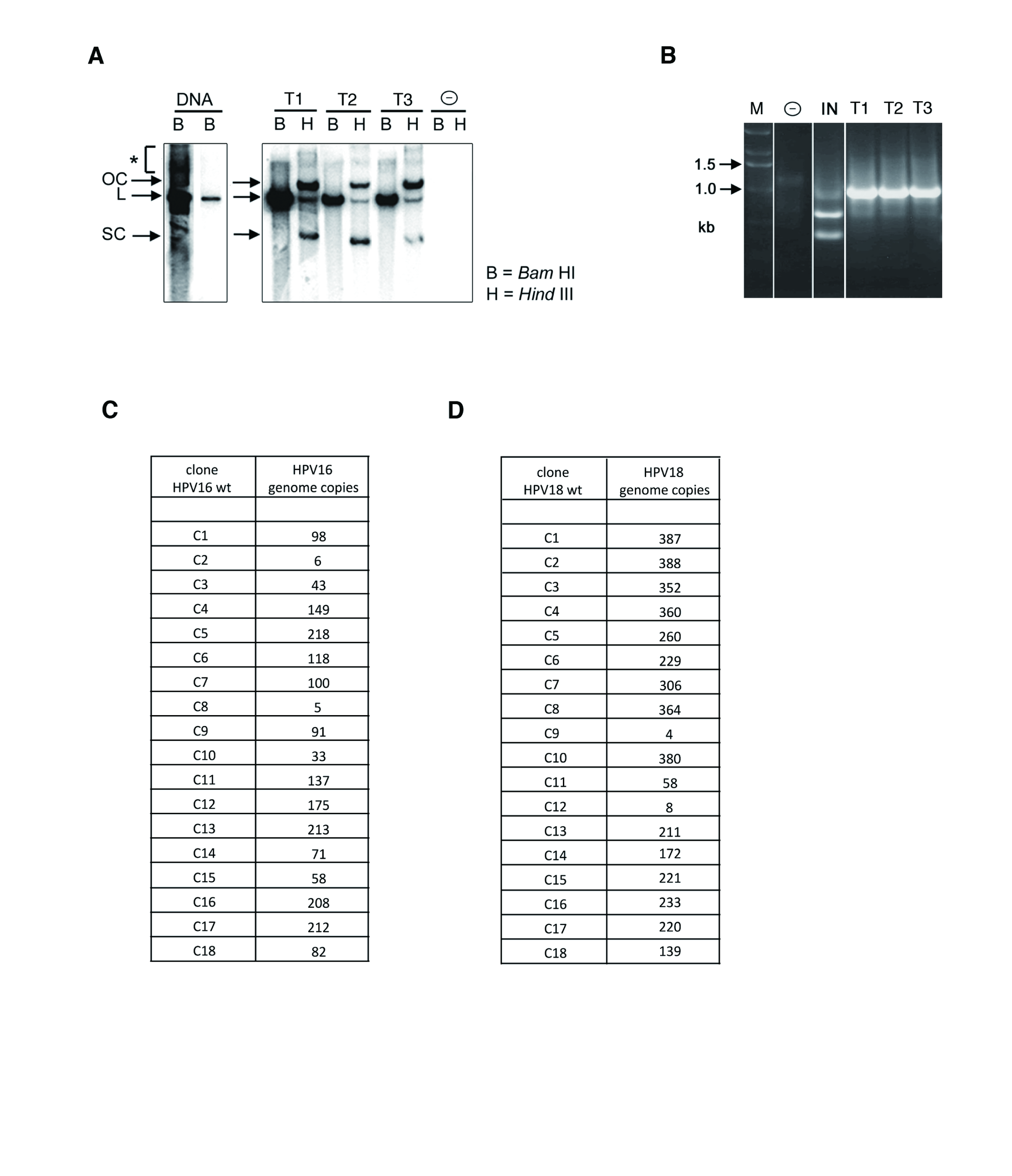

Supplement: S1 Fig — (A) Southern blot of DNA extracted from three HPV16 clonal cell populations (T1, T2 and T3) after digestion with Bam HI (B) and Hind III (H). The track labelled (-) contains DNA extracted from the parental NIKS population. Hind III digestion allowed visualisation of mostly supercoiled (SC) viral genomic DNA, along with open circle (OC) and linear (L) forms. Bam HI, which cuts once in the HPV16 genome, gave rise to a single 8kb linear band (L). Bam HI-linearized HPV-16 control genomic DNA (tracks labelled ‘DNA’) was run alongside DNA isolated from the clonal cell populations as a size marker. Slower-migrating genomic DNA visible at higher loading is marked by an asterisk. (B) To confirm that viral gene expression is primarily from episomal HPV, an APOT assay was performed on the cell lines and cell populations used in this study. The clonal cell populations (T1,T2 and T3) shown in (A) express a predominant E6/E7 transcript of approximately 1Kb, whereas cell lines with integrated HPV DNA typically contain heterogeneous transcript patterns comparable to those shown in track labelled ‘IN’. A ‘no RNA’ loading control is shown in track (-). (C & D) HPV copy number-diversity was established in 18 individual HPV16 (C) and HPV18 (D) clonal cell populations. While all cell lines harbored episomal genomes, the copy number varied between individual clones, presumably reflecting copy number variation in individual cells in the HPV16 and 18 populations. Copy number matched clones and populations were used for the comparative analysis described here. (TIF) [file ppat.1006282.s001.tif]

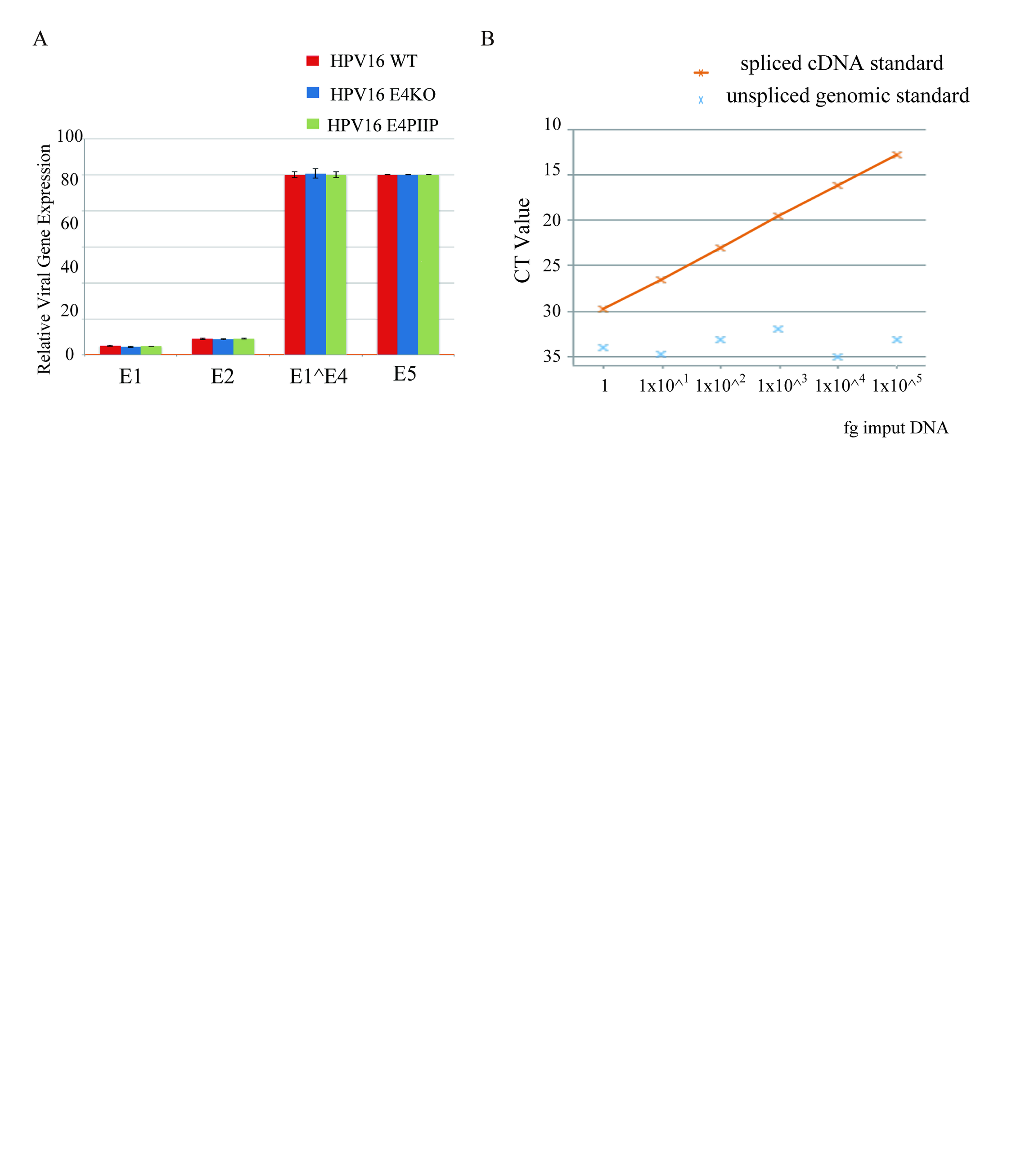

Supplement: S2 Fig — (A) Viral transcripts spanning E1, E2, or using the E1^E4 splice junction (880^3358), were quantified after reverse transcription (RT) by qPCR as described in Materials and Methods. Transcript abundance was normalized against total early transcripts measured using qPCR primers located immediately upstream of the early polyadenylation site and within the E5 ORF (columns labeled E5). In the absence of the RT step, the qPCR procedure produced negligible signal with all primer sets (mean 0.16%; SD 0.18%). No significant differences were apparent between the WT HPV16, the E4KO and E4PIIP genomes, suggesting that the presence of E4 does not affect patterns of transcription. (B) The ability of the E1^E4 primers to detect only the spliced E1^E4 transcript was assessed against a 10-fold dilution series of cloned E1^E4 cDNA (orange crosses/line) or unspliced HPV16 genomic DNA (blue crosses). The E1^E4 primers were amplified a PCR product only from spliced cDNA. (TIF) [file ppat.1006282.s002.tif]

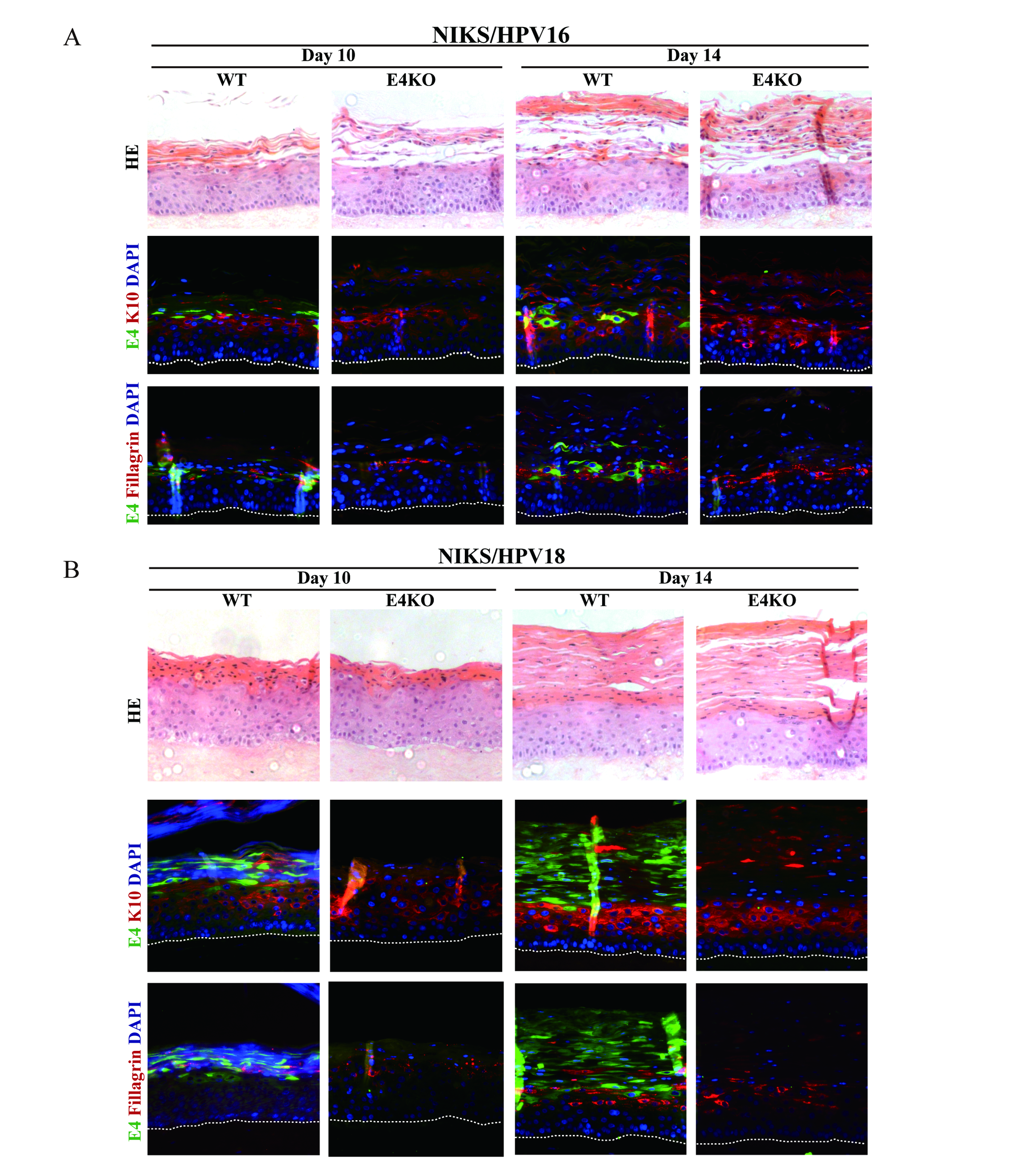

Supplement: S3 Fig — (A) Rafts prepared using HPV16 WT or E4KO genomes are shown at day 10 and day 14 after staining with Hemotoxylin and Eosin (H&E, upper panels). The middle panels show immunofluorescence stains for E4 (green) and keratin 10 (K10, red), with the lower panels showing staining for E4 (green) and filaggrin (red). Immunofluorescence images are counterstained with DAPI (blue) to allow visualization of the cell nuclei. (B) Rafts prepared using HPV18 WT or E4KO genomes and stained with H&E, or to establish the patterns of K10 and filaggrin expression as described above. (TIF) [file ppat.1006282.s003.tif]

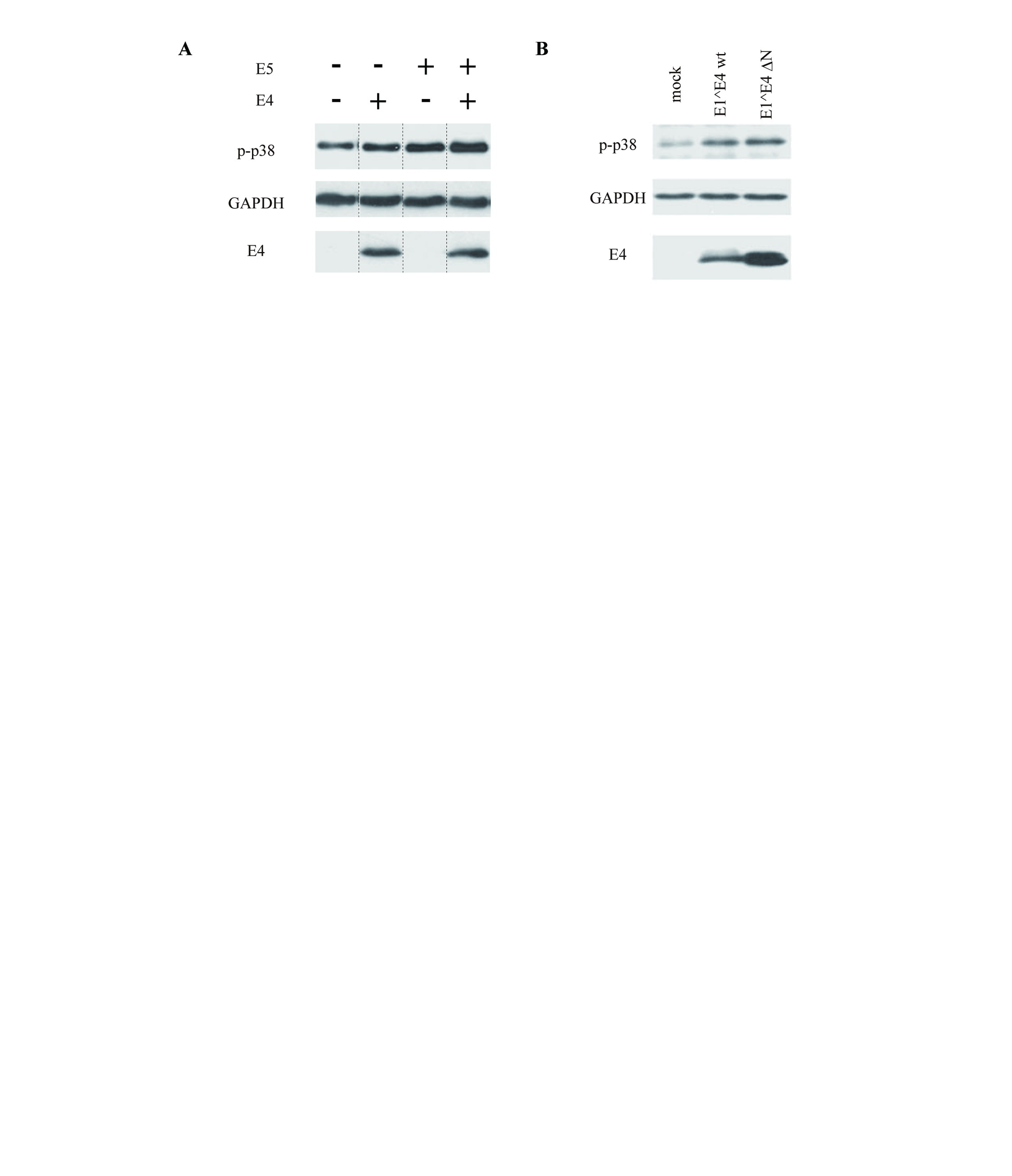

Supplement: S4 Fig — (A) 16E1^E4 was expressed from rAd16E1^E4 (tracks labelled E4+) in SiHa and SiHa_E5 cells (tracks labelled E5+). SiHa_E5 cells have been described previously [18]. Levels of activated p38 are shown in track labelled p-p38. The effects of 16E1^E4 on pERK1/2 in this system have been described previously [18]. (B) The 16 E1^E4 protein or the N-terminally deleted form of 16 E1^E4 were expressed in SiHa cells as described in Materials and Methods. Levels of activated p38 are shown in track labelled p-p38. (TIF) [file ppat.1006282.s004.tif]

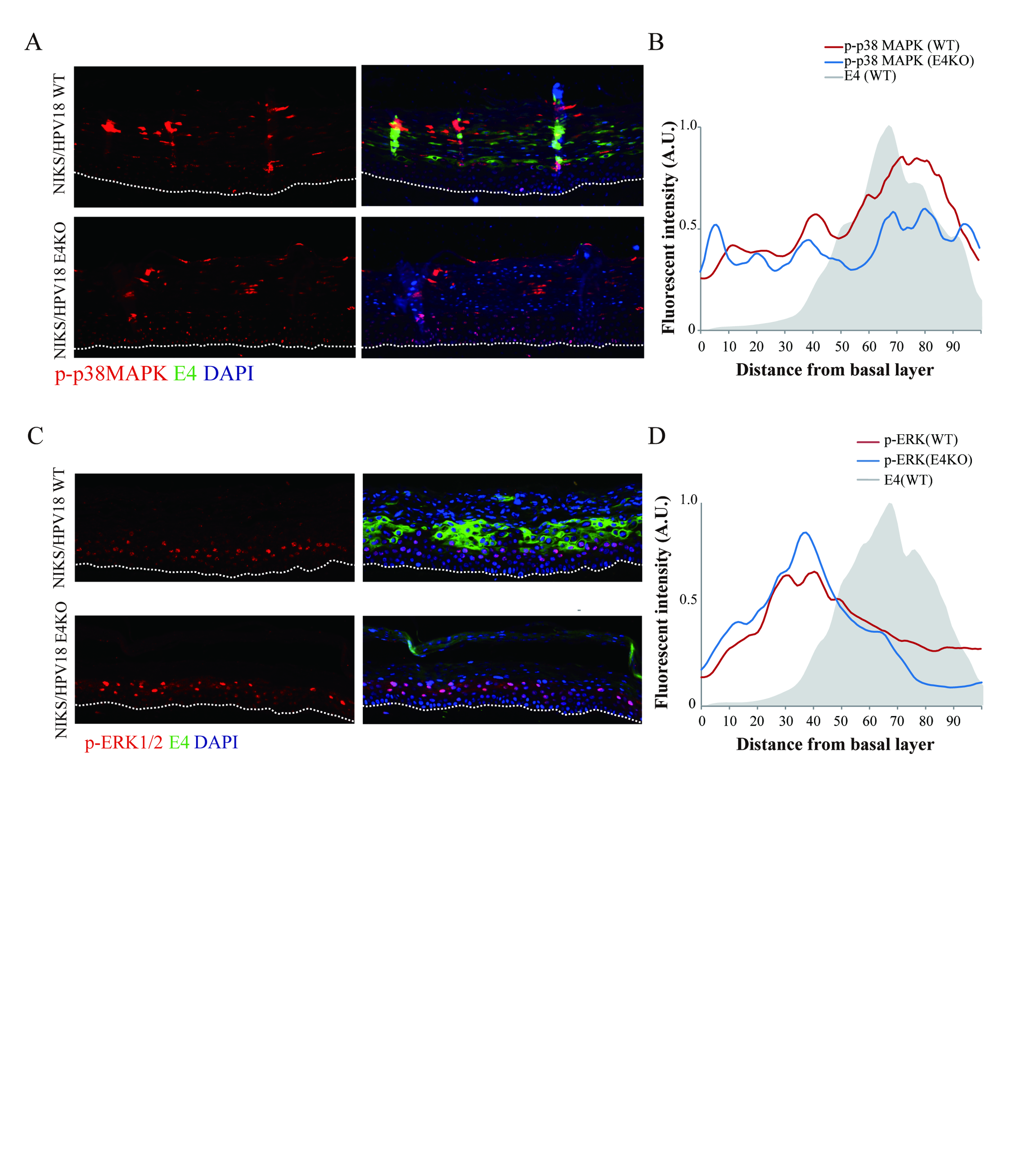

Supplement: S5 Fig — (A) Raft tissues from NIKS containing HPV18 WT or E4KO genomes were harvested at day 14 post-differentiation and stained for 18E1^E4 (green), phospho-p38 MAPK (p-p38 MAPK) (red) and DNA (blue; DAPI). A modest elevation of p-p38 MAPK staining in the upper layers of the raft is apparent in rafts generated using the WT and E4KO HPV18 genome with no significant differences between the two genomes. The dotted lines indicate the position of the basal layer. Images were captured using a 10x objective. (B) The extent and intensity of p-p38MAPK staining in the HPV18 WT and E4KO raft tissues at the 14 day time-point post differentiation was digitally scanned from the basal layer to the top of the raft tissue as described in the materials and methods. The expression of E4 in the WT raft is shown as the grey shadow. A similar level of p-p38 MAPK activity was apparent in the upper epithelial layers of rafts prepared using the WT and E4KO HPV18 genome. (C) Raft sections from HPV18 WT or E4KO genomes were harvested at 14 days post-differentiation and stained for 18E1^E4 (green), p-ERK1/2 (red) and DNA (blue; DAPI), to reveal differences in the levels of ERK1/2 activity in the mid epithelial layers. The dotted lines indicate the position of the basal layer. Images were captured using a 10x objective. (D) The extent and intensity of p-ERK1/2 staining in NIKS rafts harboring HPV18 WT or E4KO raft tissues at day 14 was examined by digitally scanning the raft tissue from the basal layer to the top of the raft. The distribution and intensity of E1^E4 staining in the WT raft is shown as a grey shadow. In contrast to HPV16, 18E1^E4 had little effect on ERK1/2, which was largely confined to the mid epithelial layers. (TIF) [file ppat.1006282.s005.tif]

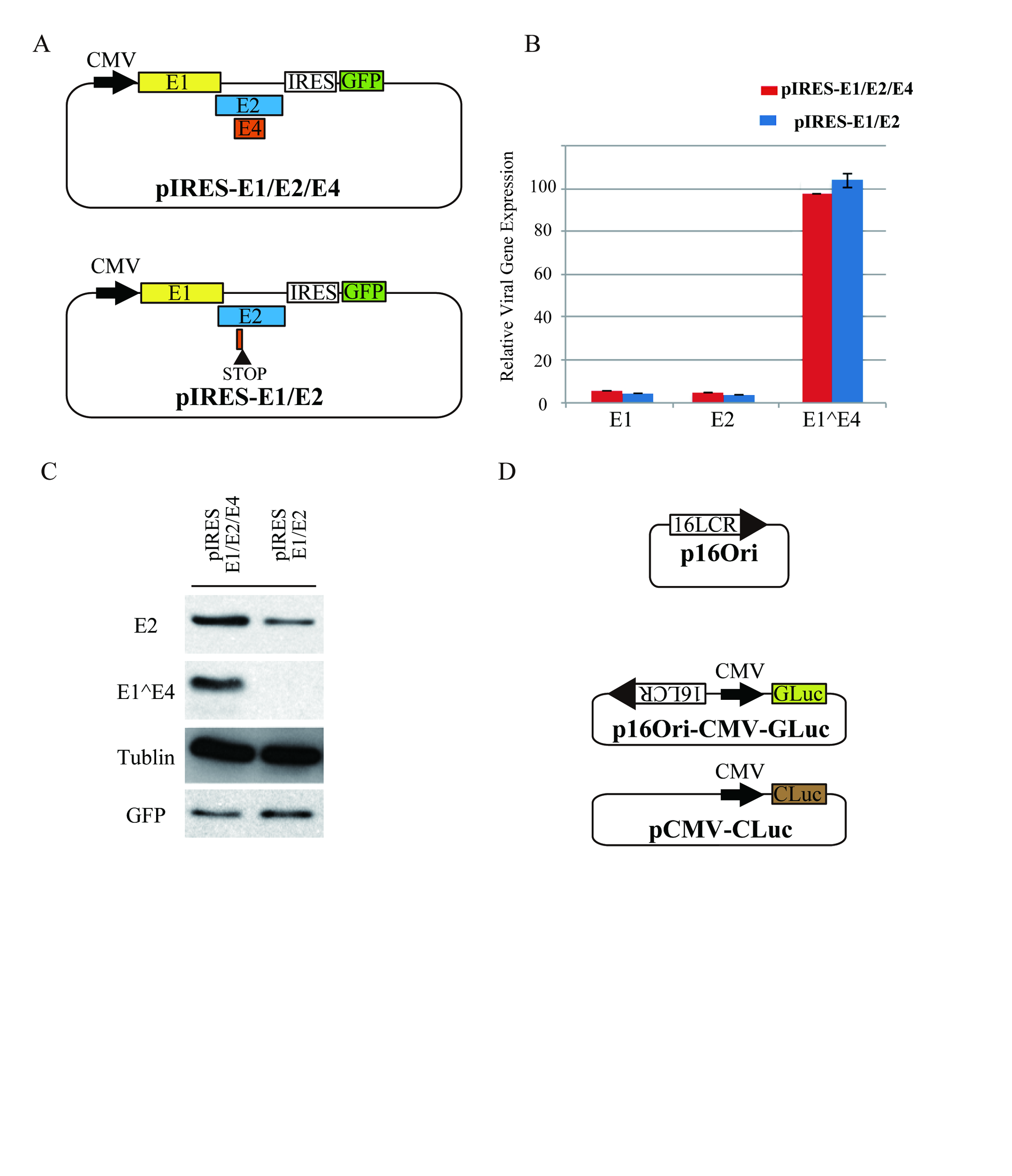

Supplement: S6 Fig — (A) Schematic representation of the E1/E2/E4 and the E1/E2 expression vectors (pIRES-16E1/E2/E4 and pIRES-16E1/E2). DNA fragment containing intact E1, E2 and E4 genes, or the E1 and E2 genes along with the E4KO gene, were cloned downstream of the CMV promoter in pIRESeGFP. The E4 ORF is contained within the E2 ORF but is translated in different reading frame from a sliced mRNA. The GFP protein is translated from the same transcripts using an internal ribosome entry sites (IRES). (B) Viral transcripts spanning E1, E2, or using the E1^E4 splice junction (880^3358), were quantified after reverse transcription (RT) by qPCR as described in Materials and Methods. As shown above, the ratio of transcripts spanning E1 or E2, or using the E1^E4 splice junction, were similar to that seen from the HPV16 WT or E4KO genomes (S2 Fig). (C) C33a cells were transfected with pIRES-16E1/E2/E4 or pIRES-16E1/E2. Cell extracts were analyzed by Western blotting with antibodies to E2, E4, tublin and GFP. In the absence of good E1 antibodies, E1 expression was visualized by RT-PCR as described in (B) above. (D) The structure of the p16 Origin plasmid (p16Ori) and the p16Ori reporter plasmids (GLuc and CLuc) are shown diagrammatically. These plasmids were used in the replication assays shown in Fig 8. (TIF) [file ppat.1006282.s006.tif]

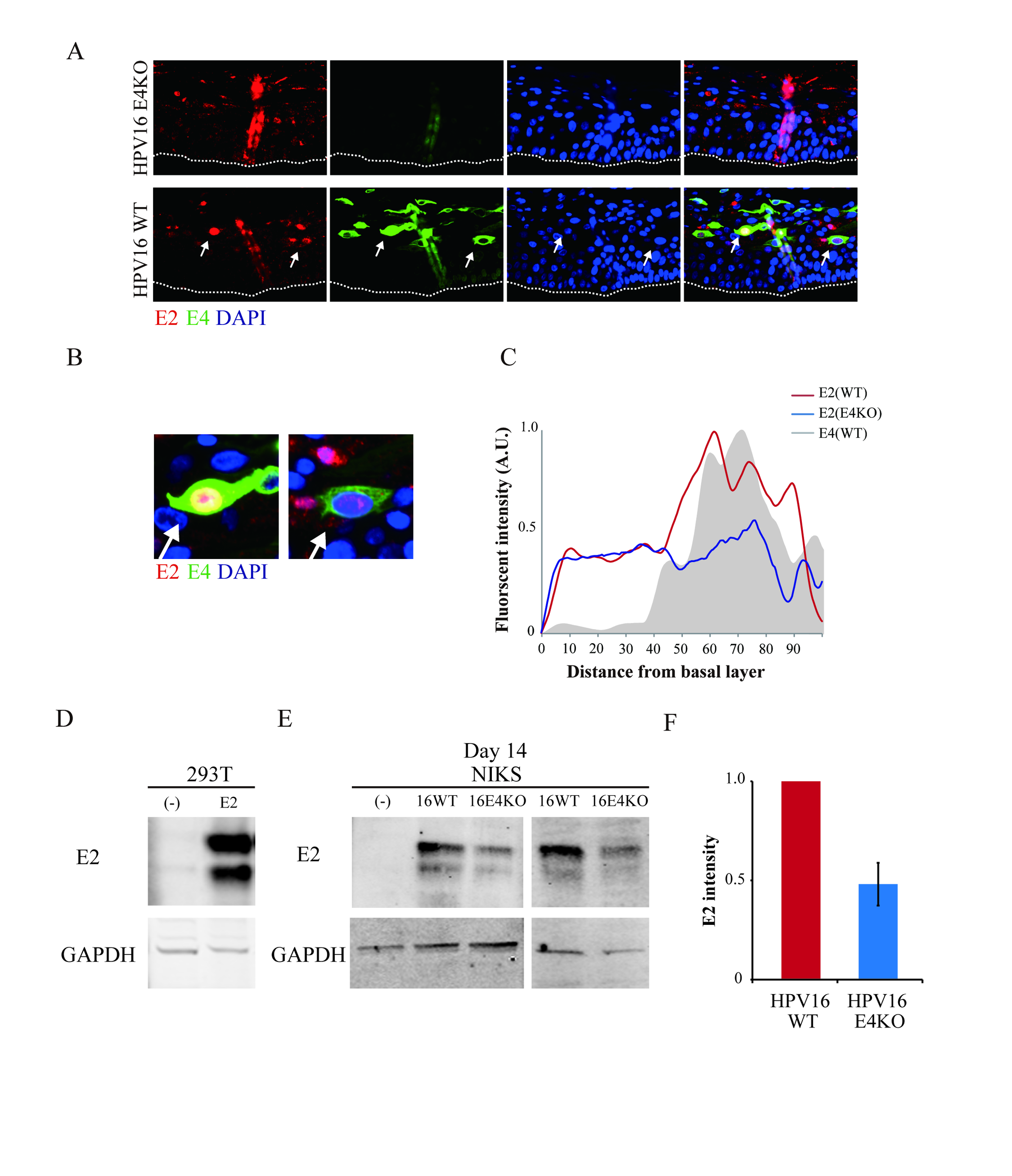

Supplement: S7 Fig — (A) Detection of E2 in HPV16 WT and E4KO rafts and correlation with E4 expression. E2 staining was carried out using the rabbit polyclonal antibody described in [35] without purification. Although some background staining was apparent with this antibody, nuclear staining, which overlapped the DAPI signal, was seen in cells positive for E4 (arrowed). Such staining was always less distinct in the 16 E4KO raft. (B) Enlarged image of cells arrowed to show the E2 (red) and E4 (green) immunofluorescence staining in the mid epithelial layers of the 16E4WT raft. (C) Digital imaging revealed a lower overall E2 signal, particularly in the mid-epithelial layers, when 16E4WT and E4KO rafts were compared. (D) Total protein extracted from 293T cells transfected with either empty or HPV16 E2 expression vector are shown in tracks labelled (-) and E2, following Western blotting using the E2 rabbit polyclonal antisera described in [35]. (E) Total protein was extracted from microdissected raft epithelium (WT or E4KO) at the 14 day time point, and the levels of E2 protein visualised by Western blotting as in (D). (F) Columns show quantitation of the Western blot signal averaged across triplicate experiments and normalized to GAPDH. Data is shown as ‘fold’ change when compared to the E2 levels observed in the presence of E4. (TIF) [file ppat.1006282.s007.tif]
